# Developmental system drift in motor ganglion patterning between distantly related tunicates

**DOI:** 10.1186/s13227-018-0107-0

**Published:** 2018-07-23

**Authors:** Elijah K. Lowe, Alberto Stolfi

**Affiliations:** School of Biological Sciences, Georgia Institute of Technology, Atlanta, GA USA

## Abstract

**Background:**

The larval nervous system of the solitary tunicate *Ciona* is a simple model for the study of chordate neurodevelopment. The development and connectivity of the *Ciona* motor ganglion have been studied in fine detail, but how this important structure develops in other tunicates is not well known.

**Methods and Results:**

By comparing gene expression patterns in the developing MG of the distantly related tunicate *Molgula occidentalis,* we found that its patterning is highly conserved compared to the *Ciona* MG. MG neuronal subtypes in *Molgula* were specified in the exact same positions as in *Ciona,* though the timing of subtype-specific gene expression onset was slightly shifted to begin earlier, relative to mitotic exit and differentiation. In transgenic *Molgula* embryos electroporated with *Dmbx* reporter plasmids, we were also able to characterize the morphology of the lone pair of descending decussating neurons (ddNs) in *Molgula,* revealing the same unique contralateral projection seen in *Ciona* ddNs and their putative vertebrate homologs the Mauthner cells. Although *Dmbx* expression labels the ddNs in both species, cross-species transgenic assays revealed significant changes to the regulatory logic underlying *Dmbx* transcription. We found that *Dmbx cis*-regulatory DNAs from *Ciona* can drive highly specific reporter gene expression in *Molgula* ddNs, but *Molgula* sequences are not active in *Ciona* ddNs.

**Conclusions:**

This acute divergence in the molecular mechanisms that underlie otherwise functionally conserved *cis*-regulatory DNAs supports the recently proposed idea that the extreme genetic plasticity observed in tunicates may be attributed to the extreme *rigidity* of the spatial organization of their embryonic cell lineages.

**Electronic supplementary material:**

The online version of this article (10.1186/s13227-018-0107-0) contains supplementary material, which is available to authorized users.

## Background

Tunicates have a long history as tractable laboratory organisms for the study of embryonic development [1]. Most tunicate larvae develop rapidly but invariantly, according to highly stereotyped cell lineages. Furthermore, many also possess highly compact genomes and are quite amenable to a wide variety of molecular assays and perturbations. More recently, tunicates have also begun emerging as model organisms for developmental neurobiology [2]. The complete connectome of the larva of the tunicate *Ciona intestinalis*, the second connectome ever mapped after that of the nematode *C. elegans* [3], revealed the synaptic connections of all 177 neurons of the central nervous system (CNS) and all 54 neurons of the peripheral nervous system (PNS) [4, 5]. With 231 total neurons (CNS and PNS combined), the *Ciona* larval nervous system is one of the smallest ever described, smaller than even the nervous system of the *C. elegans* hermaphrodite (302 neurons).

The *Ciona* connectome further revealed specific neural circuits that are conserved between tunicates and their sister group, the vertebrates, including a putative homolog of the Mauthner cell/C-start escape response circuit of fish [6]. In *Ciona,* the putative Mauthner cell (M-cell) homologs are a single pair of descending decussating neurons (ddNs), which correspond to the A12.239 pair of cells of the motor ganglion (MG), a cluster of 30 neurons that comprise a central pattern generator for the swimming behavior of the larva [7], and proposed to be homologous to a combination of the vertebrate hind brain and spinal cord [8]. The development of the ddNs and the rest of the MG have been studied in some detail, revealing gene expression patterns and transcriptional regulatory networks that are shared with hindbrain and spinal cord development in vertebrates [9–11]. These close parallels are especially striking considering the obvious, drastic reduction in size and complexity of the *Ciona* MG relative to the corresponding regions in vertebrates. However, it was found that cell fate specification and transcriptional patterning in the *Ciona* MG depends largely on cell–cell contact-dependent signaling within the neural tube by the Delta/Notch and Ephrin/Eph pathways, not on gradients of secreted, long-range morphogens as in the vertebrate spinal cord [11, 12].

Tunicates have also garnered recent attention for the fact that their extremely reduced, stereotyped cell lineages are highly conserved even between distantly related species. In spite of highly elevated mutation rates genome sequence divergence, and deep evolutionary timescales [13–16], the embryos of distantly related solitary tunicates like *Ciona* and *Molgula* (estimated divergence 390 million years apart) [15] are nearly indistinguishable [17]. We previously compared the development of the cardiopharyngeal mesoderm between *Ciona robusta* and *Molgula occidentalis* and found that there were virtually no differences in cell lineage and gene expression. However, we did find that underneath this seemingly conserved developmental program, there were considerable cryptic functional differences, resulting in *cis*-regulatory “unintelligibility” between *Ciona* and *Molgula.* In other words, homologous *cis*-regulatory elements driving identical gene expression patterns were partially or completely non-functional in cross-species transgenic assays. This phenomenon was ascribed to a particularly acute form of developmental system drift (DSD) [18]. It was recently proposed that the acute DSD observed in solitary tunicate evolution may be directly related to their mode of development, which depends primarily on invariant, contact-dependent intercellular signaling for cell fate induction. This geometric constraint would have relaxed constraints on genome evolution imposed by the relatively intricate and immutable transcriptional networks and *cis*-regulatory logics required for inductive events in species with larger, more variable embryos (i.e., the “Geometry vs. Genes” paradigm) [19].

Here we report that the phenomenon of acute DSD also extends to neurodevelopment in relatively late phases of tunicate embryogenesis. By surveying the development of the *M. occidentalis* MG, we show that transcription factor expression patterns, or “codes,” in the developing MG are identical between *Molgula* and *Ciona,* indicating near-perfect conservation of arrangement of neuronal subtypes. However, cross-species reporter assays revealed acute DSD in the regulation of *Dmbx* transcription in homologous ddN precursor cells. Our results support a “Geometry vs. Genes” model to explain the emergence of DSD in the tunicate MG, given its patterning by invariant cell–cell contacts and the parsimony of its final configuration as inferred by the *Ciona* connectome.

## Methods

### *Molgula occidentalis* genome assembly improvement

Libraries prepared for the version of the *Molgula occidentalis* genome (ELv1.2) currently available on ANISEED (https://www.aniseed.cnrs.fr/) [17, 20, 21] were reused to reassemble the genome. The original 3 DNA libraries of paired-end reads had insert sizes of 300–400, 650–750, and 875–975 base pairs (NCBI SRA ID# PRJNA253689) [17]. Reads were first filtered to a kmer coverage of 100X using the Khmer suite [22]. Assembly was done using Oases (v 0.2.08) [23] at various word lengths (k), with k = 67 being selected as the assembly to continue the downstream analyses. After reassembly (version “k67”), additional scaffolding was done using Redundans [24] producing the version “k67_R” (available at https://osf.io/3crup/). The ANISEED genome (ELv1.2) was scaffolded with Redundans as well, for comparison (version “ANISEED_R”). First redundant contigs were detected and selectively removed, and next genome fragments were joined using the previously mentioned libraries from the initial assembly. Finally, gapped regions of the scaffold were filled using these 3 paired-end libraries. After reassembly and additional scaffolding, the scaffolding quality was examined using REAPR (v1.0.17) [25] mapping all three libraries to back to the assembly.

### *Molgula occidentalis* transcriptome sequencing

Gravid *Molgula occidentalis* (Traustedt, 1883) adults were collected and shipped by Gulf Specimen Marine Lab (Panacea, FL). Eggs were fertilized as previously described [17]. *M. occidentalis* embryos and larvae were grown at ~ 24 °C and collected into three separate stage pools: 0.33 hours post-fertilization (hpf), 7, and 16 hpf. Total RNA was extracted from each pooled sample using RNAqueous Total RNA Isolation kit (ThermoFisher). PolyA+ mRNAs were selected using oligo d(T)25 magnetic beads (New England Biolabs) following two rounds of the manufacturer’s protocol. Directional RNAseq libraries were prepared according to a modified version of the protocol contained at: https://wikis.nyu.edu/pages/viewpage.action?pageId=24445095 [26]. First-strand synthesis was performed using Super Script III (ThermoFisher), primed with oligo d(T) and random hexamer primers. Second-strand synthesis was performed using dUTP instead of dTTP for directional (strand-specific) sequencing [27]. Samples were processed and ligated to NETFLEX DNA barcode adapters (BioO Scientific) for multiplex sequencing. Adapter dimers were removed using AMPure beads (Agencourt). Samples were then treated with uracil-DNA glycosylase, amplified using 12 cycles of PCR, and purified once with a minElute kit (Qiagen) and once with AMPure beads. Samples were sequenced by Illumina 2000 2 × 50 bp runs on two lanes (all three samples were multiplex-sequenced in each lane). Resulting sequences will be deposited in the NCBI SRA database (accession number pending).

### Transcriptome assembly and gene model improvement

Paired-end sequences generated above were used to generate gene model. Reads were quality filtered/trimmed using trimmomatic (v0.33) [28] and the following parameters “MINLEN:25, and sliding window of 4 with a minimum score of 5 (ILLUMINACLIP:TruSeq3-PE.fa:2:30:10 SLIDINGWINDOW:4:5 MINLEN:25).” Each of three embryonic stage libraries (0.33, 7, and 16 hpf) were individually mapped to the various genome versions using hisat2 (v 2.1.0) [29], with the default parameters. The sam files generated from hisat2 were then sorted and converted to bam using samtools (v 1.5) [30] and merged using PicardTools with java version 1.8 (http://broadinstitute.github.io/picard/). The merged bam file was then processed using StringTie (v 1.3.3b) [31] to create gene models. Gene models were then extracted using the gffread utility from cufflinks [32] and evaluated using BUSCO for completeness [33]. Transcripts/gene models have been deposited at OSF (https://osf.io/3crup/).

### Fertilization, dechorionation, and electroporation of embryos

*M. occidentalis* eggs were fertilized, dechorionated, and/or electroporated as previously described [17]. *Ciona robusta* (*intestinalis* type A) adults were collected and shipped by M-REP (San Diego, CA) and eggs dechorionated, fertilized, and electroporated as previously described [34]. *Dmbx cis*-regulatory DNAs from two *Molgula* species (*M. occidentalis* and *M. oculata*) were cloned intro reporter plasmids as illustrated in supplemental sequences files (Additional file 1). *Cirobu.Dmbx*>*Unc*-*76::Venus* and *Cirobu.Fgf8/17/18*>*H2B::mCherry* reporter plasmids were published previously [10, 11].

### mRNA probe synthesis and in situ hybridization

Templates for antisense riboprobes for in situ hybridization were amplified by PCR or SMARTer 3’/5’-RACE (Clontech) from cDNA libraries or from genomic DNA (see Additional file 1 for details on each sequence). Template sequences were cloned either using TOPO-TA cloning (ThermoFisher) into pCRII dual promoter vector, or using restriction enzyme cloning into pCiProbe (see Additional file 1) linearized NotI-EcoRI. *In vitro* transcription of labeled riboprobes and two-color fluorescent in situ hybridization were performed as previously described [35].

## Results and discussion

### *M. occidentalis* transcriptome assembly and gene prediction

We recently sequenced the genomes of 3 species in the genus *Molgula*: *M. occidentalis, M. oculata,* and *M. occulta* [17], which can be browsed freely on the Tunicate molecular biology database ANISEED (https://www.aniseed.cnrs.fr/) [21]. Of these, *M. occidentalis* emerged as a valuable species for comparative studies of tunicate development, mainly because their zygotes can be transfected with plasmid DNAs via electroporation, much like the major tunicate laboratory model species in the *Ciona* genus (*C. intestinalis, C. robusta,* and *C. savignyi)*. To help establish additional molecular tools for developmental studies in *M. occidentalis,* we assembled a transcriptome based on RNAseq of 3 stages of embryonic development. These were then used to predict a new set of gene models found in the *M. occidentalis* genome sequence.

**Fig. 1:**
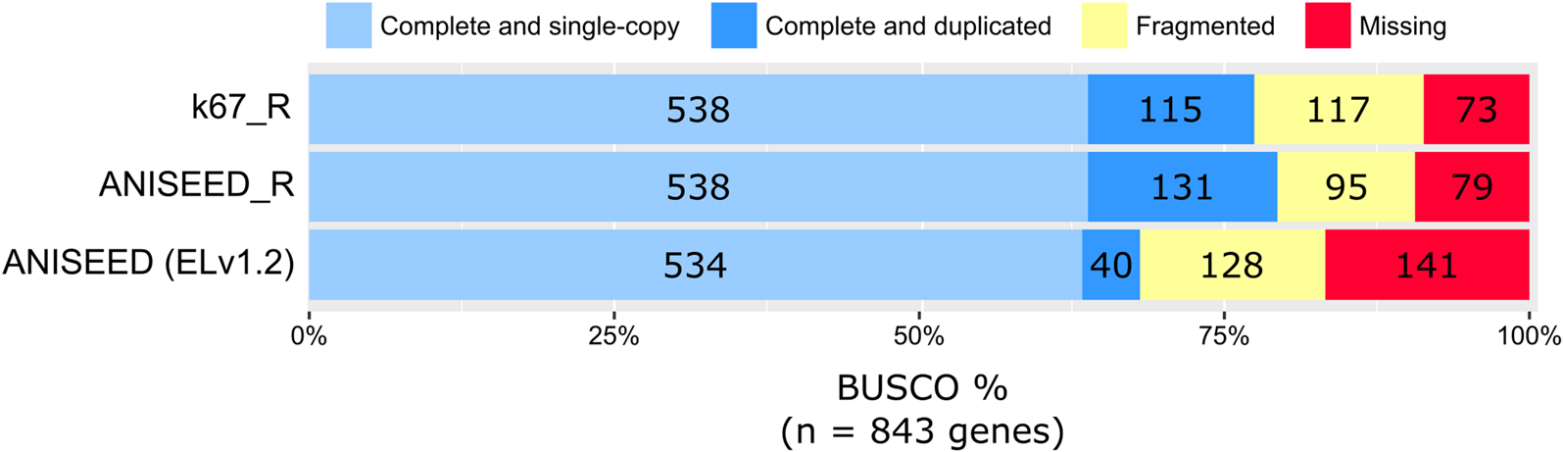
Benchmarking Universal Single-Copy Orthologs (BUSCO) in *M. occidentalis.* BUSCO was used to asses gene model predictions based on the previously published and released *M. occidentalis* genome assembly (ANISEED ELv1.2) and on the re-assemblies from this study (k67_R and ANISEED_R). BUSCO is used as a measure of completeness of gene model sets predicted from genome assemblies, searching for 843 highly conserved genes that should be present in single copies in > 90% of metazoan genomes. There are five categories of gene recovery: complete and single-copy, complete and duplicated, fragmented, and missing. Complete genes are those that preserve ~ 95% of the gene length. Single-copy genes are those with only one copy in the gene model set and duplicated genes are those with multiple copies identified either by gene duplication or assembly errors. Fragmented genes are those recovered with less than 95% of the gene length, and missing genes are those that are not found to be present at all

To do this, we also reassessed our previous *M. occidentalis* genome assemblies. In previous assemblies, some of the low-coverage regions were removed using khmer to reduce assembly fragmentation [22]. To preserve the information contained in these low-coverage regions, we re-assembled the genome from the original raw sequencing reads (see “Methods” section for details). While this new assembly initially had an N50 of only 519 bp and over 680,000 scaffolds, additional scaffolding decreased the number of scaffolds to 30,188, with 20,616 of those (68%) being over 1 kb in length (Table 1). As a result of this better scaffolding, the N50 also increased from 519 to 18,312. The gap filling process decreased the number of missing bases (Ns) from 5,510,404 to 189,152. The end result is the “k67_R” genome assembly version, which we have deposited at OSF (https://osf.io/3crup/). While these procedures improved the genome assembly statistics, we wanted to ensure that the additional gene content was still preserved. To check this, we mapped mRNA reads and produced gene models to be tested using BUSCO [33]. This allowed us to compare our new gene models against a defined collection of highly conserved sequences expected to be present in all metazoans, as a measure of assembly completeness. Our BUSCO results (Fig. 1) indicated that fewer reference genes were missing (73 missing out of 843 total) in the new “k67_R” assembly than in the ANISEED ELv1.2 version (141/843 missing) or even a version of the ANSEED assembly with additional scaffolding performed (“ANISEED_R,” 79/843 missing). This indicates that these new versions of the genome assembly and their corresponding gene models should enhance the identification and cloning of sequences to use as molecular tools for *M. occidentalis.*

**Table 1:** *M. occidentalis* genome reassembly statistics

Assembly version	k67_R	ANISEED_R	ANISEED (ELv1.2)
Total length (bp)	207,168,883	242,783,314	262,547,660
N’s	189,152	885,908	33,473,590
Scaffolds	30,188	16,148	51,761
Scaffolds ≥ 1 kb	20,616	13,751	21,251
Longest scaffold (bp)	250,303	235,023	229,829
N50 (bp)	18,312	35,542	26,298

### Gene expression patterns in the *M. occidentalis* motor ganglion

**Fig. 2:**
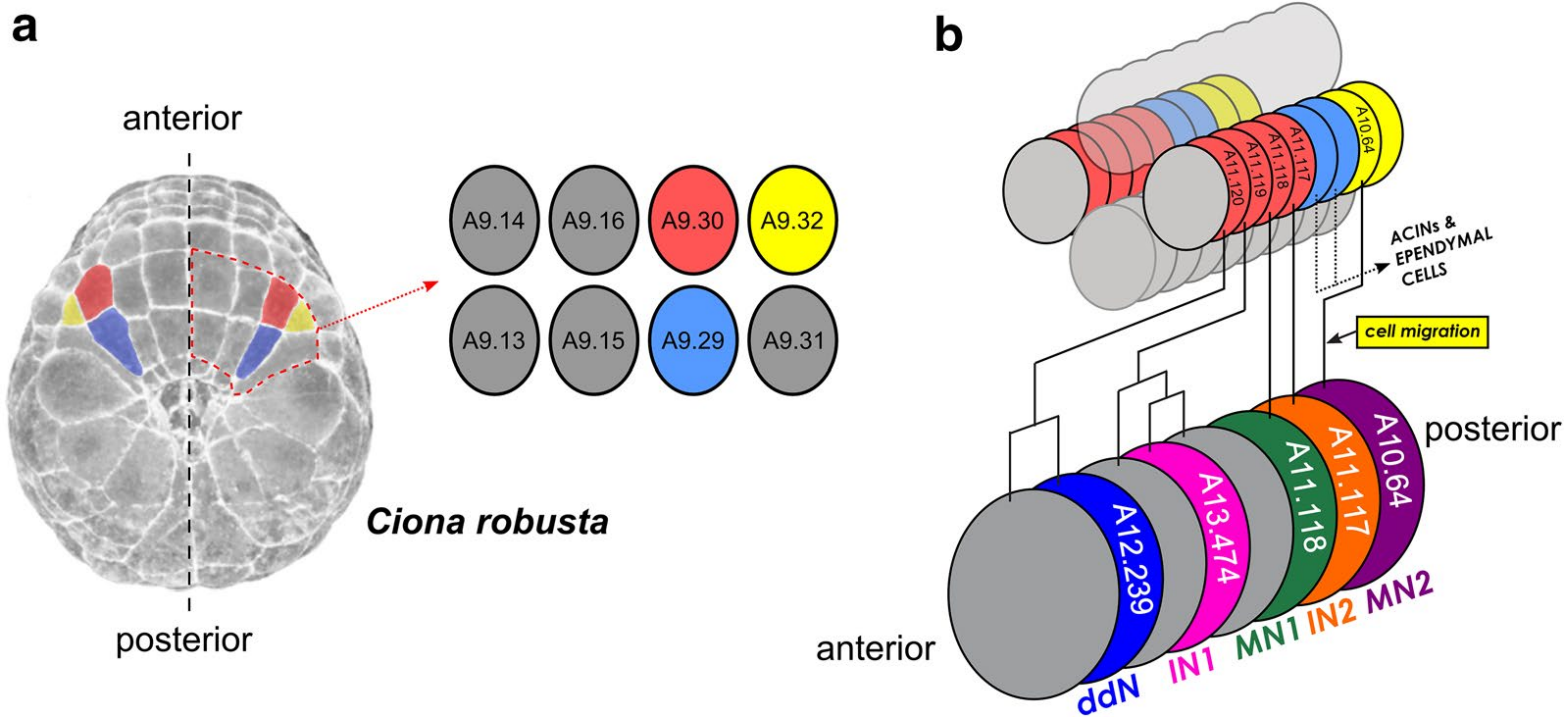
Motor ganglion lineages and neuron subtype configuration in *Ciona.* **a** Dorsal view of a late gastrula-stage *Ciona robusta* embryo highlighting in false color the bilaterally symmetric right/left pairs of blastomeres in the vegetal neural plate that give rise to the “core” Motor Ganglion and Anterior Caudal Inhibitory Neurons (ACINs) of the nerve cord: A9.30 (red), A9.32 (yellow), and A9.29 (blue). Black dashed line indicated embryo midline. Red dashed outline denotes the right vegetal neural plate annotated in the inset diagram, which indicates the exact cell identities according to the established cell lineage nomenclature [64]. Embryo image adapted from the Four-dimensional Ascidian Body Atlas website (FABA, http://chordate.bpni.bio.keio.ac.jp/faba/1.4/top.html) [65]. **b** Diagram representing a section of the dorsal hollow neural tube derived from the neural plate after the process of neurulation. Cells of the neural tube are color-coded according to their lineages using the same color scheme as the neural plate diagram in (**a**). The neural tube is comprised exactly of four single-file rows of cells: 1 dorsal row, 2 lateral rows, and 1 ventral row. Cells of the lateral rows contributing to the bilaterally symmetric motor ganglion are: A11.117-A11.120, derived from A9.30, and A10.64, derived from A9.32. The A9.29 lineage, which gives rise to ACINs and ependymal cells posterior to the MG, is not illustrated in great detail, for the sake of simplicity. At bottom is a diagram of the 5 core MG neuron types. Black lines indicate their descent patterns from those cells at top. ddN = descending decussating neuron, IN1 = interneuron 1, IN2 = interneuron 2, MN1 = motor neuron 1, MN2 = motor neuron 2. Non-neuronal cells are gray. Cells are identical on both left and right sides

The *Ciona* MG is comprised of 22 neurons and includes a “core” MG of 5 morphologically and molecularly distinct left/right pairs of neurons [4]. In the remainder of this study, we will only refer to the neurons on one side, for simplicity. The core MG is derived from the A9.30 and A9.32 blastomeres of the neural plate (Fig. 2a) that will give rise to cells along the lateral rows of the neural tube after neurulation and neural tube closure [36–39]. At the level of the MG, the neural tube of the *Ciona* embryo is formed by only four single-file rows of cells oriented along the anterior–posterior (A–P) axis: a dorsal row (roof plate), a ventral row (floor plate), and two neurogenic lateral rows from which most of the core MG neurons are specified (Fig. 2b). Ascending contralateral inhibitory neurons (ACINs) derived from the A9.29 blastomeres [40] have been traditionally excluded from the MG based on their more posterior location at the base of the tail. However, these are likely indispensable cogs in the MG central pattern generator, driving left/right alternation of tail contractions during swimming by glycinergic neurotransmission [7]. Remaining MG neurons are poorly studied, and their development is largely unknown. In this study, we focused on the “core” MG neurons, those derived from the A9.30 and A9.32 lineages, because these lineages have been the most thoroughly studied MG lineages [9, 10, 39]. Expression patterns of developmental regulators and provisional gene regulatory networks have been documented in these lineages [9], and the induction events responsible for specifying the 5 distinct types of neurons in this core MG have also been elucidated [10, 11]. Thus, the MG is a perfect starting point for a comparative study on the evolution of neurodevelopment in tunicates.

**Fig. 3:**
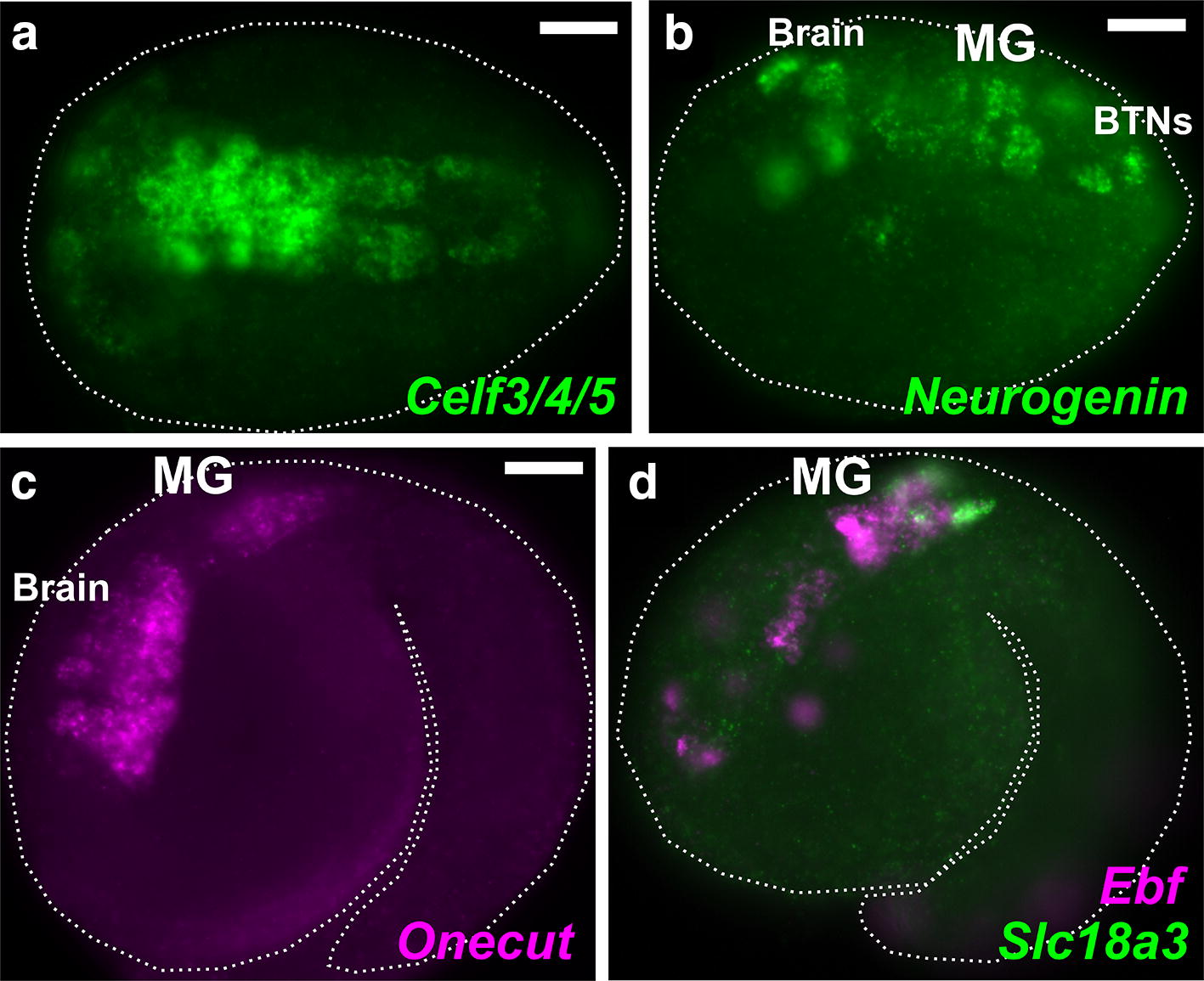
The developing central nervous system of *Molgula occidentalis.* **a** Dorsal view of a neurula stage *Molgula occidentalis* embryo. Green shows fluorescent in situ hybridization for *Celf3/4/5* (also known as *Etr*-1) transcription. **b** In situ hybridization for *Neurogenin* in initial tailbud embryo, showing transcription in the embryonic brain, motor ganglion (MG), and bipolar tail neurons (BTNs). **c** In situ hybridization for *Onecut,* expressed in brain and MG. **d** Two-color fluorescent in situ hybridization showing co-expression of *Ebf* (magenta) and *Slc18a3* (also known as *VAChT*, green) in the MG. Embryo outlined by dotted lines. Anterior is to the left in all images. All scale bars = 25 µm

We previously showed that the overall development of the *M. occidentalis* embryo is very similar to the development of the *Ciona* embryo [17]. An in-depth study of the cardiopharyngeal mesoderm (B7.5 cell lineage) revealed perfect conservation of precise cell divisions and fate specification events, with only minimal differences in morphogenesis and timing of gene expression. However, other tissues beyond the cardiopharyngeal mesoderm were not surveyed. In this study, we sought to extend our understanding of tunicate evolution by comparing the developing nervous systems of *M. occidentalis* and *C. robusta* (*intestinalis* type A). In situ mRNA hybridization (ISH) for neural marker *Celf3/4/5* (formerly *Etr*-*1*) in *M. occidentalis* neurula embryos revealed the CNS developing from the lateral rows of the dorsal hollow neural tube, as in *Ciona* [41] (Fig. 3a). Later, at tailbud stages, ISH for the neuronal transcription factors *Neurogenin, Onecut,* and *Ebf* revealed ongoing neuronal specification in the brain, MG, and bipolar tail neurons of the tail (Fig. 3b–d), also identical to the expression of these regulators in *Ciona* embryos [41, 42]. Two-color ISH of *Ebf* and the cholinergic marker *Slc18a3* (also known as *vesicular acetylcholine transporter,* or *VAChT*) [43] revealed the earliest differentiating neurons of the larval CNS, the motor neurons of the MG (Fig. 3d). Given that no significant differences were revealed between *Molgula* and *Ciona* using these broad markers of neural fate, we focused our attention specifically to the developing MG, where we would be able to analyze more subtype-specific gene expression and cell fates.

In *C. robusta*, the expression patterns of mRNAs encoding transcription factors are dynamically regulated in the developing MG [9, 44]. Some of these patterns comprise a conserved “code” of homeodomain-containing genes that are differentially expressed along the dorsal–ventral (D-V) developing spinal cord of vertebrate embryos [11]. However, in the developing *Ciona* MG, the neurogenic domain of neural tube is restricted to the single-file lateral rows of cells. Therefore, the D-V code of the vertebrate spinal cord is transposed along the A-P axis in *Ciona.* From these neural progenitors expressing different combinations, or codes, of these homeodomain proteins, the 5 distinct neuronal subtypes of the *Ciona* MG are born. We sought to characterize the expression patterns of homologous genes in the developing MG of *M. occidentalis.* We were able to identify these readily by performing BLAST against our transcriptome assembly, which revealed clear orthologs for the following genes: *Mnx, Vsx, Islet, Nk6, Pax3/7,* and *Dmbx.* An additional gene *Lhx3/4.a* had already been identified and characterized in our previous study [17].

### Motor neuron and interneuron identities

**Fig. 4:**
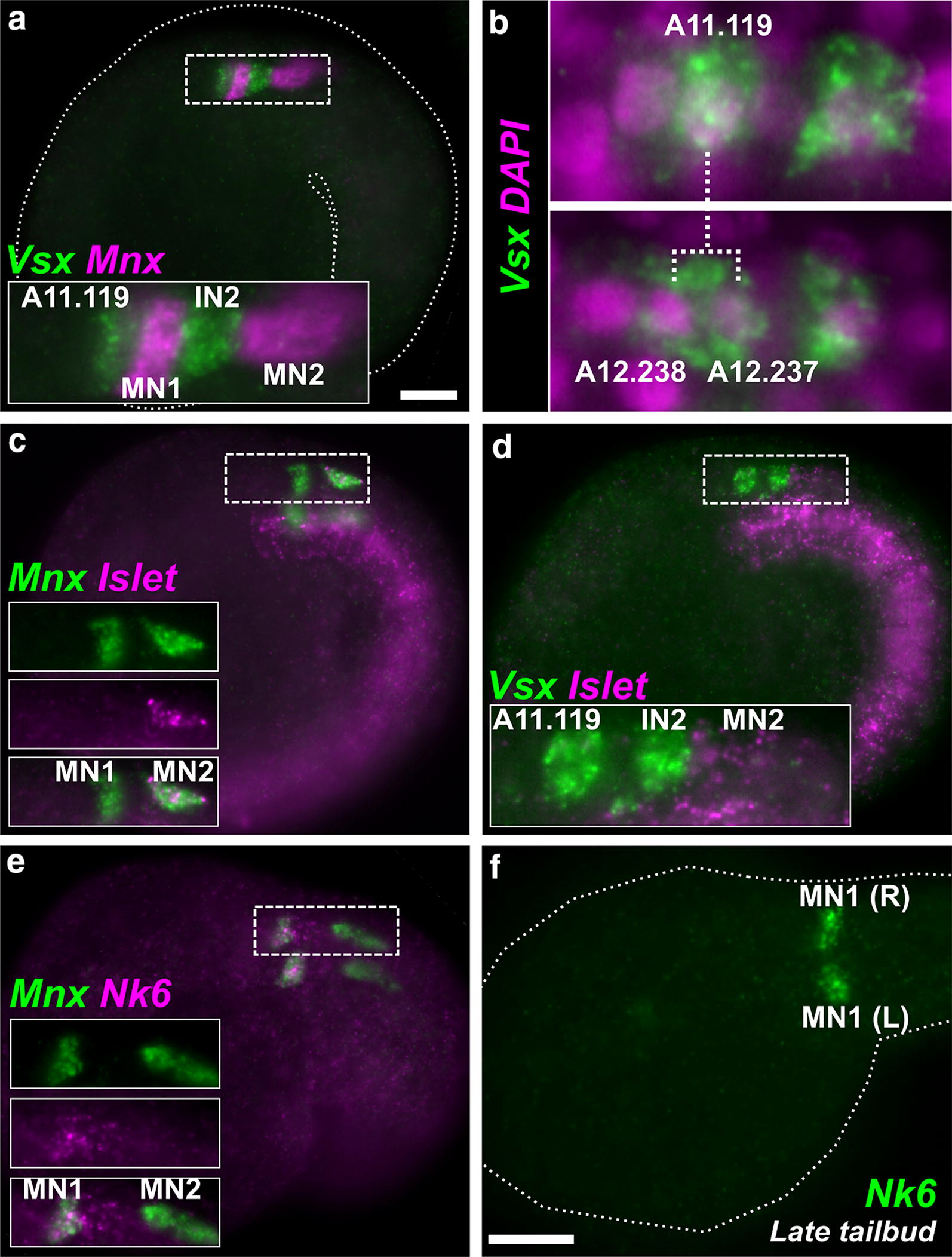
Motor neurons and interneurons in the MG. **a** Lateral view of mid-tailbud stage *Molgula occidentalis* embryo (dotted outline). Two-color fluorescent in situ hybridization reveals alternating expression of interneuron marker *Vsx* (green) in undifferentiated A11.119 interneuron progenitor cell and interneuon 2 (IN2) and motor neuron marker *Mnx* (magenta) in motor neurons 1 and 2 (MN1, MN2). Only one side of embryo is shown. Inset is a magnified view of dashed box. **b** In situ hybridization for *Vsx* (green) in embryos at two successive stages. *Vsx* is initially activated in A11.119 (top panel), which divides and gives rise to daughter cells A12.238 and A12.237, both of which continue to express *Vsx* at this stage. These data confirm that *Vsx* is activated in the A11.119 cell in *M. occidentalis*, which is not the case in *Ciona* (see text for details). Nuclei are counterstained with DAPI (magenta). **c** Two-color fluorescent in situ hybridization for *Mnx* (green) and MN2 marker *Islet* (magenta), revealing identities of MN1 (*Mnx*+*/Islet*-*)* and MN2 (*Mnx*+*/Islet*+*)* cells. Boxed area magnified in insets. **d** Two-color fluorescent in situ hybridization for *Vsx* (green) and *Islet* (magenta). Only one side of embryo is in focus. **e** Two-color fluorescent in situ hybridization for *Mnx* (green) and MN1 marker *Nk6* (magenta), confirming identities of MN1 (*Mnx*+*/Nk6*+*)* and MN2 (*Mnx*+*/Nk6*-*).* **f** *Nk6* in situ hybridization (green) in late tailbud embryo, showing sharpened expression in MN1 cells on both sides of the MG (R = right side and L = left side). All scale bars = 25 µm

In *Ciona,* 4 of the 5 core MG neuron subtypes are marked by either *Mnx* or *Vsx* [9, 10]. These are orthologs of conserved transcription factors that specify motor neurons (HB9, MNR2, etc.) or interneurons (Chox10, CHX10, Ceh-10, etc.), respectively. Two-color ISH revealed a neatly alternating *Vsx*-*Mnx*-*Vsx*-*Mnx* pattern on one side of the developing MG of *M. occidentalis,* around 8.5 hours post-fertilization (hpf) (Fig. 4a). This alternating pattern closely mirrors the alternation of motor neuron (MN) and interneuron (IN) subtypes in the *Ciona* MG: IN1-MN1-IN2-MN2. In the *Ciona* MG, the latter three are the first MG neurons to differentiate, corresponding to cells A11.118 (MN1), A11.117 (IN2), and A10.64 (MN2), which are consecutively arrayed with no intervening cells between them. This appears to be the case in *M. occidentalis* also. However, in *Ciona* there are additional non-neuronal cells intercalated between IN1 and MN1, as a result of continued proliferation in the anterior MG that ultimately gives rise to IN1 (see Fig. 2b). In *Ciona, Vsx* expression is restricted to post-mitotic interneurons and is not observed in A11.119, the grandmother cell of IN1 [10]. In *M. occidentalis,* the lack of any discernible gap between the anterior-most *Vsx*+ and *Mnx*+ cells at first suggests that *Vsx* transcription starts in the A11.119 progenitor cell itself, a clear example of transcriptional “priming” of cell fate [45]. Indeed, we observed *Vsx* expression in two anterior cells when observed at slightly later stages, suggesting that A11.119 divided after the onset of *Vsx* activation (Fig. 4b). Together with evidence for earlier *Dmbx* expression in the A11.120 progenitor cell (see below), these data suggest that the expression of certain MG neuron subtype-specific transcription factors is already primed in *M. occidentalis* MG progenitors. This heterochronic shift may be related to the ~ 10% faster development of *Molgula* relative to *Ciona* [17].

### *Islet* expression reveals MN2 (A10.64 cell)

From the *Mnx* ISH, MN2 appeared to be the posterior-most cell of the core MG. The identity of this cell was confirmed by two-color ISH with *Islet,* a marker of MN2 fate in *Ciona* (Fig. 4c, d). In *Ciona,* MN2 is the only core MG neuron that is not derived from the A9.30 lineage. Long identified as the A10.57 cell derived from the A9.29 lineage, MN2 was recently revealed in fact to be the A10.64 cell of the A9.32 lineage instead and ultimately derived from the A8.16 neuromesodermal lineage that also gives rise to the secondary tail muscles of the larva [39]. Despite its origin from further posterior in the embryo, MN2 becomes associated with the MG thanks to a dramatic, anterior migration along the outside of the neural tube, leapfrogging over the entire A9.29 lineage [39]. The migrating MN2 is elongated along the A-P axis due to the extension of a leading edge that ultimately contacts the A9.30 lineage. Upon contacting the A9.30 lineage, MN2 extends its axon posteriorly, retaining an elongated cell body and forming *en passant* synapses with the dorsal band of muscle cells down the length of the tail [46]. In *M. occidentalis,* the elongated morphology of MN2 was readily apparent by ISH, in which fluorescent signal fills most of the cell bodies to reveal their shapes. Therefore, we conclude that, in *M. occidentalis,* the specification and morphogenesis of MN2 are highly conserved.

### *Nk6* expression is refined and restricted to MN1

While MN2 controls tail contractions in a graded manner [47], the other major MN in *Ciona* is MN1, the A11.118 cell. This neuron was shown to form large, leaf-like (“frondose”) motor endplates at the base of the tail [10]. While MN2 s are proposed to exert graded motor control during maneuvering, the all-or-none flexions of the tail that drive swimming are thought to be triggered instead by MN1 s. This cell is characterized by *Mnx* expression without co-expression of *Islet.* In *Ciona,* MN1 is also marked by late, sustained expression of *Nk6* close to hatching, even though this gene is expressed broadly throughout the posterior A9.30 lineage earlier in development [11]. In *M. occidentalis,* we found this also to be true. *Nk6* expression was seen in A11.119, A11.118 (MN1), and A11.117 (IN2) at the mid-tailbud stage (Fig. 4d), but later was seen only in a single pair of cells in the embryo, presumably MN1s (Fig. 4e). This further confirms the highly conserved nature of the posterior MG and the specification of MN1 by sustained *Nk6* expression.

### Subfunctionalization of *Lhx3/4* paralogs

**Fig. 5:**
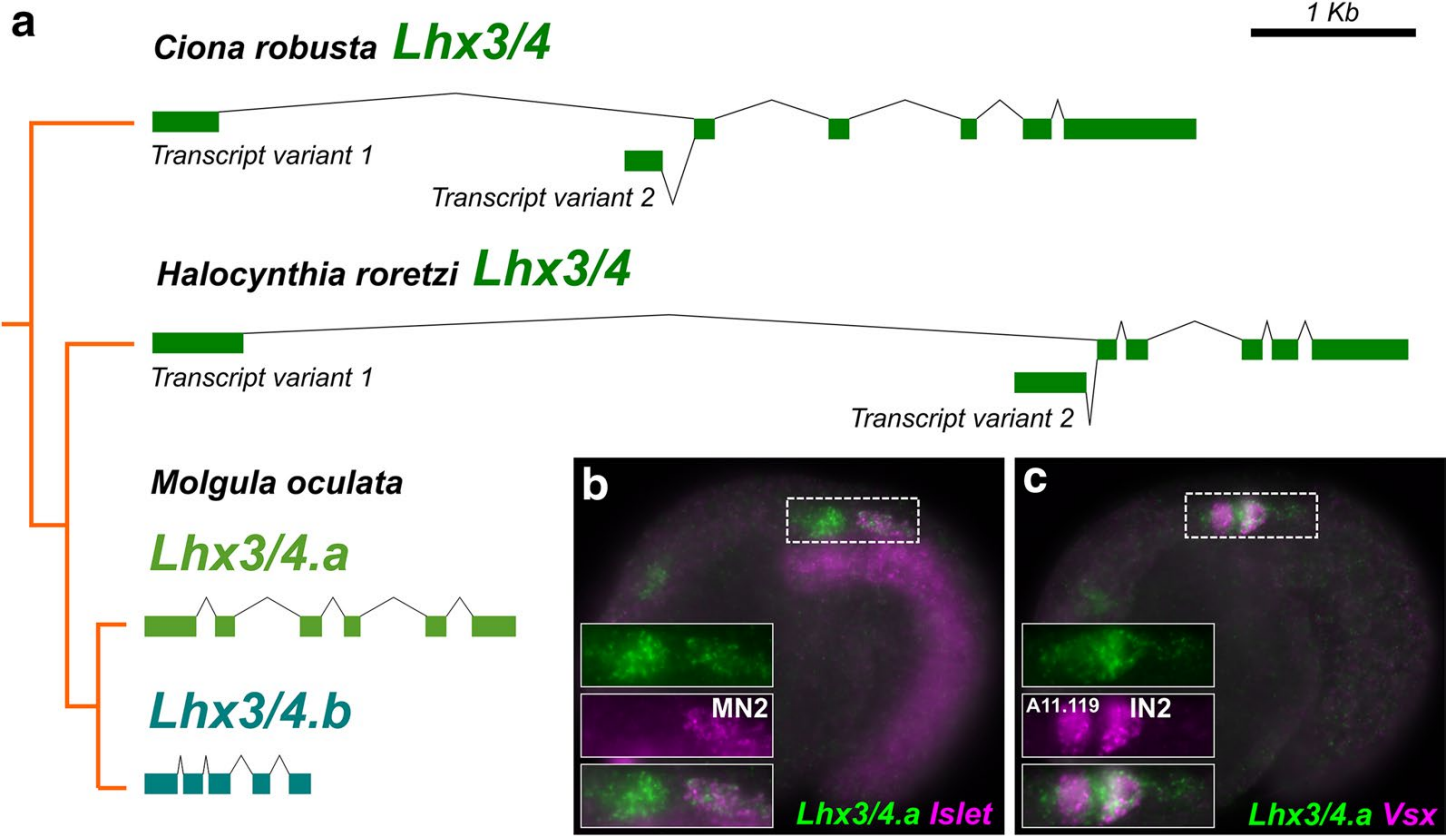
*Molgula*-specific duplication and subfunctionalization of *Lhx3/4.* **a** Diagram depicting *Lhx3/4* genes in the genomes of *Ciona robusta, Halocynthia roretzi,* and *Molgula oculata,* with their phylogenetic relationships indicated by the orange tree. All diagrams are at the same scale (scale bar is at top right). Exons indicated as colored bars, introns are depicted by crooked lines. Both *Ciona* and *Halocynthia* have a single *Lhx3/4* gene that is transcribed from two distinct promoters to give rise to two transcript variants. In both species, variant 2 is involved in vegetal pole patterning in the early embryo, while variant 1 is involved in motor ganglion patterning. In all three *Molgula* species sequenced, *Lhx3/4* has been duplicated, giving rise to paralogs *Lhx3/4.a* and *Lhx3/4.b.* Loci from *M. oculata* are shown here (instead of *M. occidentalis*) because its genome is the least fragmented *Molgula* genome. *M. occidentalis Lhx3/4* genes are similar in structure to their *M. oculata* orthologs, but assembly errors near the 3’ end of *M. occidentalis Lhx3/4.b* (not shown) prevented us from using sequences from this species in our diagram. **b** Two-color fluorescent in situ hybridization for *Lhx3/4.a* (green) and *Islet* (magenta) showing co-expression in MN2. **c** Two-color fluorescent in situ hybridization for *Lhx3/4.a* (green) and *Vsx* (magenta) showing strong co-expression in IN2, and diffuse *Lhx3/4.a* expression throughout the MG

In *Ciona* and the stolidobranch tunicate *Halocynthia roretzi,* a single *Lhx3/4* is transcribed as two alternative isoforms, transcript variants 1 and 2, originating from alternate promoters (Fig. 5a) [48, 49]. In both *Ciona* and *Halocynthia,* transcript variant 1 is expressed in MG precursors, while transcript variant 2 is expressed in early vegetal pole cells and is required for endoderm and cardiopharyngeal mesoderm specification [49, 50]. We previously found that in all three *Molgula* genomes sequenced to date, the ancestral tunicate *Lhx3/4* gene was duplicated, resulting in two paralogs, termed *Lhx3/4.a* and *Lhx3/4.b* (Fig. 5a) [17]. In *M. occidentalis,* their expression patterns and putative functions appear to mirror those of the two different transcript variants identified in *Ciona/Halocynthia: Lhx3/4.a* is expressed in the MG, while *Lhx3/4.b* is only expressed in the early vegetal pole cells [17]. *Lhx3/4.a* is the more conservative paralog, retaining an ancestral C-terminal peptide motif, which has been lost in *Lhx3/4.b* [17]. Given that *Halocynthia* is more closely related to *Molgula* than to *Ciona* [51], this suggests a *Molgula*-specific duplication of *Lhx3/4*, followed by subfunctionalization through partitioning of the expression domains of the ancestral transcript variants in addition to protein-coding changes.

Here we looked more closely at the expression of *Lhx3/4.a* in the developing MG of *M. occidentalis*. In *Ciona, Lhx3/4* is initially expressed in the posterior MG (MN1, IN2, and more weakly in MN2) and later in IN1 [9, 44]. In *Halocynthia, Lhx3/4* was also detected earlier in development, in the A9.30 blastomere [52]. In *M. occidentalis,* we found *Lhx3/4.a* expressed throughout the entire MG at the mid-tailbud stage, though more strongly in MN1 and IN2 (Fig. 5b, c). Higher expression in these cells is shared with *Ciona* and *Halocynthia,* and the weaker expression in the anterior MG may reflect fading expression from earlier stages as in *Halocynthia.* Based on these results, we propose that *Lhx3/4.a* has, for the most part, conserved the MG-specific expression pattern and putative function of the ancestral *Lhx3/4* gene, more specifically those of its transcript variant 1.

### *Pax3/7* and *Dmbx* delineate the descending decussating neurons (ddNs)

The anterior-most neuron in the core MG of *Ciona* is the descending decussating neuron (ddN), corresponding to the A12.239 cell. The recently mapped *Ciona intestinalis* connectome revealed that these neurons are likely homologous to giant reticulospinal Mauthner cells (M-cells) of vertebrates and situated in a neural circuit that is topologically very similar to M-cell escape circuits [6]. In *Ciona,* the ddN is specified by a gene regulatory network involving the transcription factors Pax3/7 and Dmbx [11]. To summarize, Pax3/7 is sufficient and necessary for the specification of the A11.120 progenitor, the mother cell of the ddN, and directly activates *Dmbx* expression in the posterior daughter cell of A11.120, which becomes the ddN (A12.239). *Dmbx* in turn encodes a repressor that mediates an FGF-regulated switch for differentiation [11]. Upon differentiating, ddNs project their nascent axons across the midline, perpendicular to the A-P axis. Thus, as their name implies, they are the only core MG neurons that decussate.

**Fig. 6:**
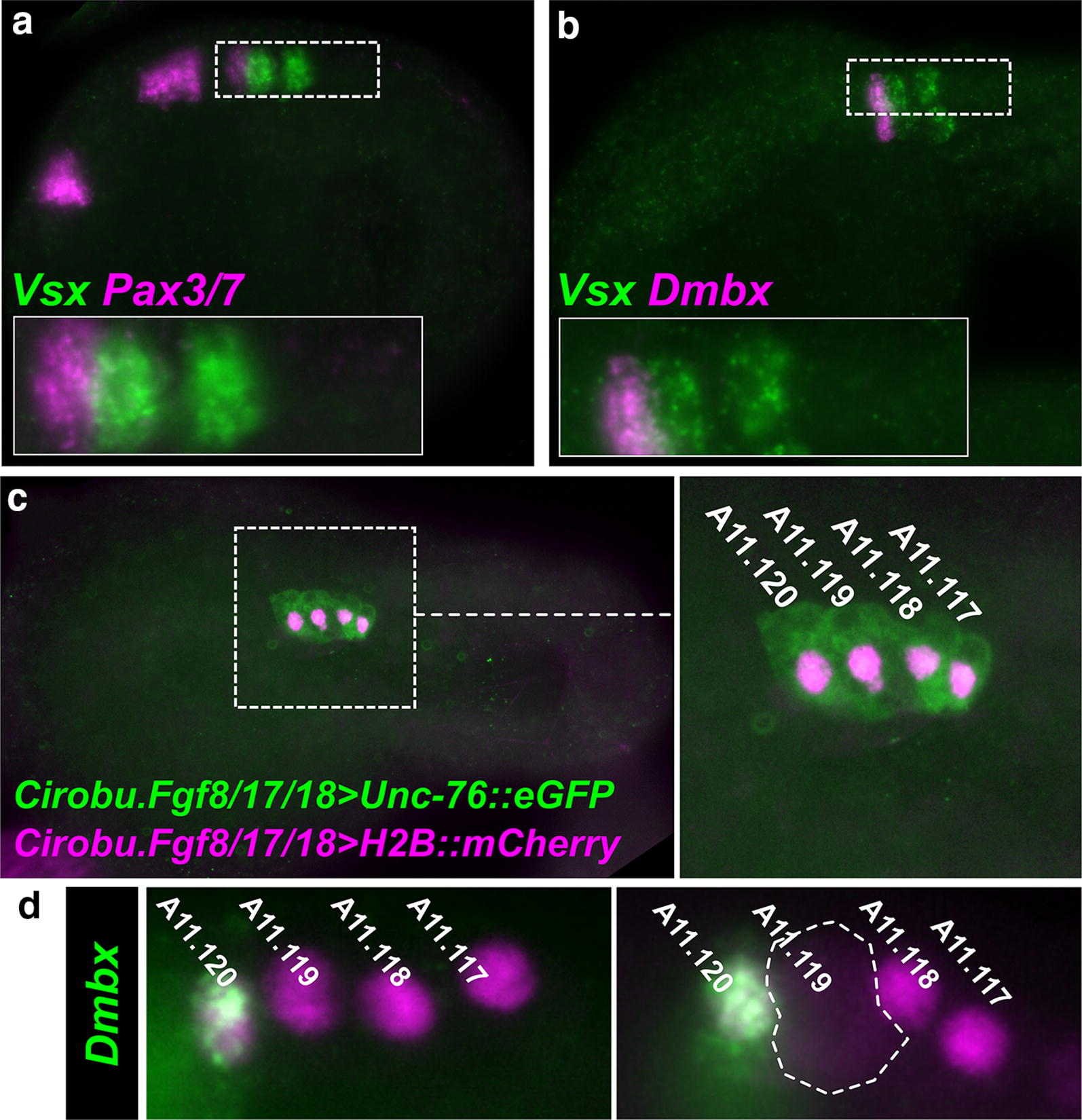
*Pax3/7* and *Dmbx* activation in A11.120. **a** Two-color fluorescent in situ hybridization for *Vsx* (green) and *Pax3/7* (magenta) showing expression of *Pax3/7* anterior and adjacent to *Vsx* expression, shown in Fig. 4 to correspond to the A11.119 cell. *Pax3/7* is thus deduced to be expressed in the sister cell of A11.119, A11.120. This is identical to its expression pattern in *Ciona.* **b** Two-color fluorescent in situ hybridization for *Vsx* (green) and *Dmbx* (magenta) also showing expression of *Dmbx* in A11.120 like *Pax3/7.* This is different from the situation in *Ciona,* in which *Dmbx* is detected only later, in the descending decussating neuron (ddN, equivalent to A12.239, the posterior daughter cell of A11.120). **c** *M. occidentalis* early tailbud embryo electroporated with *Ciona robusta* (*Cirobu) Fgf8/17/18* reporter plasmids, labeling the A9.30 lineage on one side, with H2B::mCherry-labeled nuclei in magenta and Unc-76::eGFP-labeled cell bodies in green. Inset at right showing higher magnification of boxed area, showing all four descendants of A9.30, arrayed from anterior to posterior. **d** In situ hybridization of *Dmbx* coupled to immunostaining of beta-galactosidase (magenta nuclei) driven by *Cirobu.Fgf8/17/18*>*nls::lacZ* reporter plasmid, revealing that *Dmbx* is strongly activated in the A11.120 cell, the mother cell of the ddN. Right panel shows A11.119 entering mitosis indicated by nuclear envelope breakdown and diffusion on nuclearly localized beta-galactosidase (dashed outline). A11.119 appears to divide before the other cells in the MG at this stage, which is also the case in *Ciona.* This further confirms the precocious activation of *Vsx* in this cell relative to *Ciona* (see Fig. 4)

We therefore sought to characterize the expression patterns of *Pax3/7* and *Dmbx* in *M. occidentalis.* ISH revealed *Pax3/7* and *Dmbx* expression in a single cell in the MG, just anterior to the *Vsx*-expressing cell identified as A11.119 (Fig. 6a, b). These data indicate that *Pax3/7* and *Dmbx* are co-expressed in A11.120, the mother cell of the ddN. While *Pax3/7* expression in A11.120 is shared between *Molgula* and *Ciona, Dmbx* expression in *M. occidentalis* appears to be shifted earlier relative to mitotic exit of the neuron. In *Ciona,* transcription of *Dmbx* is not detected in the A11.120 cell but is activated later, specifically in the ddN, by combinatorial action of Pax3/7 (inherited from the mother cell), and Neurogenin [11].

To confirm that *Dmbx* is expressed in the A11.120 cells of *M. occidentalis,* we sought to determine its expression pattern at cellular resolution. We turned to electroporation to transfect embryos with a reporter construct that could selectively label the A9.30 lineage and allow us to combine ISH with immunofluorescence. To this end, we used the *Fgf8/17/18* promoter from *C. robusta* [9] to drive expression of histone 2B::mCherry (*Cirobu.Fgf8/17/18*>*H2B::mCherry*) in the A9.30 lineage of electroporated *M. occidentalis* embryos. Activity of this reporter plasmid was relatively weak and infrequent in *M. occidentalis* embryos, but managed to label the 4 cells of the developing MG of *M. occidentalis,* equivalent to the cells A11.120, A11.119, A11.118, and A11.117 (from anterior to posterior) (Fig. 6c), and ISH + immunofluorescence revealed *Dmbx* expression in A11.120 at this stage (Fig. 6d). Taken together, these data indicate a *M. occidentalis*-specific priming of *Dmbx* transcription in the progenitor of the ddN, similar to the priming of *Vsx* in the progenitor of IN1 in this species as well (see above).

### Conserved morphogenesis and axon projection of ddNs in *M. occidentalis*

**Fig. 7:**
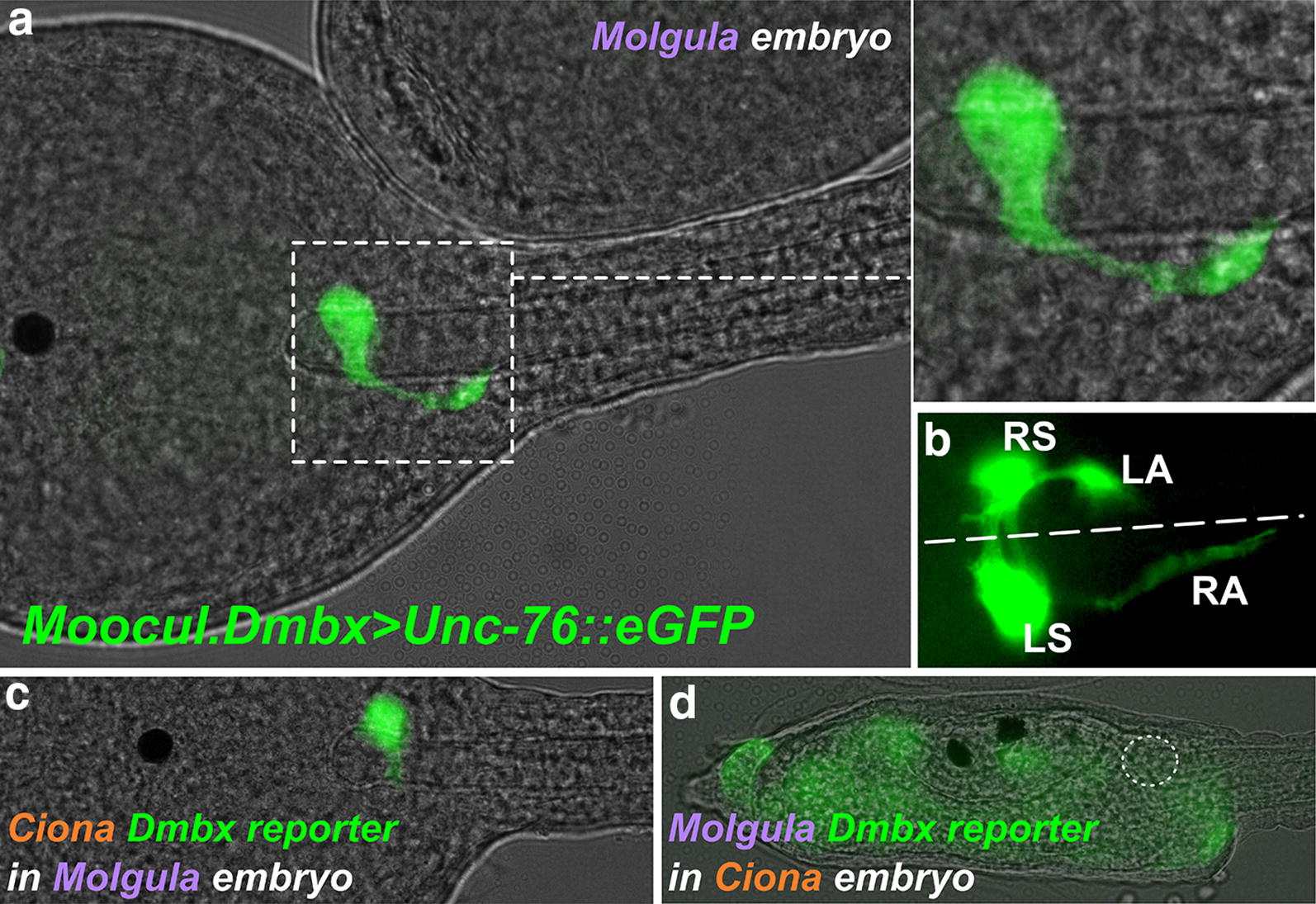
*Dmbx* reporter assays reveal conserved morphogenesis of the ddN and asymmetric unintelligibility of *cis*-regulatory sequences. **a** Dorsal view of a *Molgula occidentalis* embryo electroporated with a *Molgula oculata (Moocul) Dmbx*>*Unc*-*76::eGFP* reporter plasmid, specifically labeling (in green) a developing descending decussating neuron (ddN) on one side of the embryo. Inset at top right shows magnified view of boxed area, showing the conserved axon trajectory of the ddN, which is initially perpendicular to the anterior–posterior axis and then turns 90 degrees to continue posteriorly toward the tail. **b** Another embryo electroporated with *Moocul.Dmbx*>*Unc*-*76::eGFP,* this time labeling both right and left ddNs. Both axons cross the midline (dashed line) to continue down toward the tail on the other side. RS: right soma, RA: right axon, LS: left soma, LA: left axon. **c** *M. occidentalis* embryo electroporated with *Ciona robusta* *Dmbx*>*Unc*-*76::Venus* reporter, which is activated precisely and robustly in the ddN (green). **d** *Ciona robusta* embryo electroporated with *Molgula oculata Dmbx*>*Unc*-*76::eGFP* reporter, which is not active in this species’ ddN (approximate location indicated by dashed circle). Note that non-specific expression (green) can be seen in other unrelated tissues and cell types (palps, mesenchyme, etc.), suggesting that transcription/translation of GFP was not globally affected (see text for details)

Given the unique morphology and axon trajectory of the ddNs in *Ciona* and their likely homology to M-cells in a conserved, pan-chordate escape network [6], we asked whether their morphogenesis is conserved in *M. occidentalis.* To label differentiated ddNs, we electroporated *M. occidentalis* embryos with a *Dmbx* reporter plasmid containing ~ 1.4 Kb of genomic DNA sequence 5’ to the ATG corresponding to the predicted start codon of the *Dmbx* mRNA from the related species *M. oculata* (Additional file 1), fused to the *Unc*-*76::eGFP* fluorescent reporter gene to label axons uniformly [53]. This construct was sufficient to label the ddNs of *M. occidentalis* (Fig. 7a, b). Unc-76::eGFP expression in the ddNs revealed a polarization along the medial–lateral axis, perpendicular to the A-P, resulting in their unique axon trajectory initially straight across the midline, then abruptly turning 90° to descend along the outside of the neural tube toward the tail. This closely mirrors the processes of axonogenesis and axon guidance that result in the conserved axon trajectory of the ddNs in *Ciona* [10]. This suggests that ddN morphogenesis is an ancient and highly conserved process in tunicates, which also shares features with M-cell morphogenesis in vertebrates [54].

### Cross-species *Dmbx* reporter assays reveal asymmetric developmental system drift (DSD)

In our comparison of cardiopharyngeal development between *Ciona* and *Molgula,* we discovered pervasive, acute developmental system drift (DSD) [18] that has resulted in cross-species incompatibility of orthologous *cis*-regulatory DNAs that regulate identical gene expression patterns in the tunicate cardiopharyngeal mesoderm [17]. Here we tested whether DSD might also underlie the highly conserved gene expression patterns seen in the developing MG. We found that the previously identified *C. robusta Dmbx* driver [10] was sufficient to activate reporter gene expression in *M. occidentalis* ddNs (Fig. 7c). However, the *M. oculata Dmbx* reporter plasmid, used to label *M. occidentalis* ddNs, was non-functional in *Ciona* embryos (Fig. 7d). Thus, there is an asymmetry in the intelligibility of *Dmbx cis*-regulatory sequences: the *Ciona cis*-regulatory sequence works in *Molgula,* but not vice versa. This incompatibility is probably not due to basal promoter incompatibility since widespread non-specific *M. oculata Dmbx*>*GFP* expression was clearly visible in a variety of cell types in *Ciona* (Fig. 7d). Furthermore, the incompatibility is also not likely due to differences in *trans*-splicing (resulting in a failure to translate the fluorescent reporter protein), since we found that *Dmbx* is also *trans*-spliced in *M. occidentalis* (Additional file 1: Fig. S1).

One possible explanation for this asymmetry is that, in *Molgula, Dmbx* is initially activated in the A11.120 cell, the mother cell of the ddN (see Fig. 6d). In *Ciona*, *Dmbx* is only detected later, in the post-mitotic ddN [10], and is activated by a combination of Neurogenin and Pax3/7 and depends critically on the presence of a Pax3/7 binding site located in a *cis*-regulatory element that controls *Dmbx* transcription in the ddN [11]. Therefore, it is possible that an alternate *trans*-regulatory logic drives the earlier activation of *Dmbx* that we observe in *Molgula,* considering that *Pax3/7* and *Dmbx* are transcribed simultaneously in *M. occidentalis* MG, not sequentially as in *Ciona*. The required factors for this earlier Pax3/7-independent activation of *Dmbx* in *M. occidentalis* might therefore be absent from the *Ciona* MG, resulting in lack of activation of the *Molgula* reporters in *Ciona.* In contrast, we show that both *Pax3/7* (see Fig. 6a) and *Neurogenin* (see Fig. 3b) expression patterns are conserved in *M. occidentalis,* which would allow for proper activation of the *Ciona Dmbx* reporter plasmid in this species.

Alternatively, the incompatibility we observed may not be due to divergent *trans*-regulatory logic. In fact, we previously showed that the trans-regulatory logic of cross-species incompatibility of *Mesp* reporter plasmids between *Ciona* and *Molgula* is in part due to coevolution of the Tbx6-related transcription factor and its target *Mesp* promoter [17]. Thus, it is possible that activation of *Dmbx* by Pax3/7 and Neurogenin is conserved in *Molgula* and that the cross-species incompatibility of our *Dmbx* reporter plasmids is instead due to significant *cis/trans* coevolution between these trans-activating factors and the *Dmbx cis*-regulatory sequence. Regardless of the exact underlying molecular mechanism, the asymmetric intelligibility of *Dmbx cis*-regulatory sequences we have uncovered is yet another example of acute DSD between *Ciona* and *Molgula*, which stands in stark contrast to the overall conservation of MG development that we have documented in this study.

## Conclusions

**Fig. 8:**
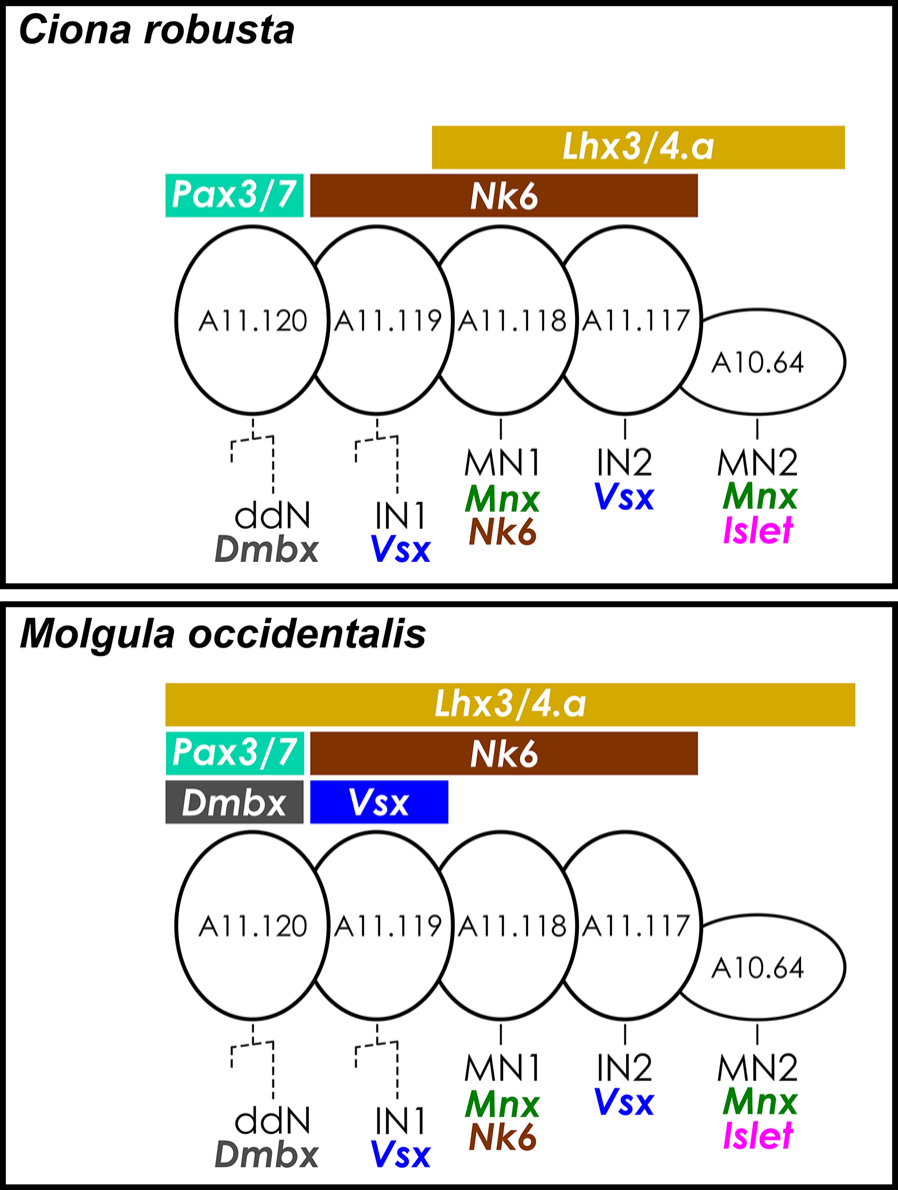
Summary of transcription factor expression patterns in *Ciona* and *Molgula.* Comparison of transcription factor expression patterns in the developing MG of *Ciona robusta* and *Molgula occidentalis,* summarizing the findings of the current study. *C. robusta* patterns are from references [10, 11, 35]. Gene expression domains during the early patterning of MG progenitors at the mid-tailbud stage are indicated by colored bars. MG neuron subtype-specific gene expression (ddN, IN1, MN1, IN2, MN2) indicated below. Dashed lines represent additional cell divisions before final specification and differentiation of ddN and IN1, which were resolved in *C. robusta* but not in *M. occidentalis.* The major differences observed in *M. occidentalis* are: early expression of *Dmbx* in A11.120 and *Vsx* in A11.119, and broader expression of *Lhx3/4.a* relative to *Lhx3/4* in *C. robusta*

Here we have documented the deep conservation of regulatory gene expression patterns that establish the precise configuration of neuronal subtypes in the MG of the tunicate larva (summarized in Fig. 8). Our data suggest that the circuitry of the MG, as revealed by the recently completed *C. intestinalis* connectome [4], may be as rigidly conserved as the embryonic development of the larva itself. We did not observe any indication that certain MG neuron subtypes identified in *Ciona* are missing in *M. occidentalis,* or that additional subtypes (not observed in *Ciona*) are specified in *M. occidentalis.* Not only does there appear to be a 1-to-1 correspondence of core MG neurons between *Ciona* and *M. occidentalis,* their position in the MG is not altered. The only differences observed are precocious transcription of certain markers in *M. occidentalis* (e.g., *Vsx, Dmbx*), which may be related to the faster developmental rate of this species relative to *Ciona* spp. These observations suggest that the MG of the solitary tunicate larva has changed very little in nearly 400 million years, the estimated time of divergence between *Ciona* and *Molgula* [15]. As such, the tunicate larval MG likely represents a minimal but ancient and exquisitely adapted central pattern generator for swimming behavior.

Our finding of acute, asymmetric DSD of *Dmbx* regulation hints at a possible cause for divergence in the regulation of seemingly identical gene expression patterns. In this case, both *C. robusta* and *M. occidentalis* specify a single pair of *Dmbx*+ ddNs. However, precocious transcriptional priming of *Dmbx* in the ddN progenitor (A11.120) in *M. occidentalis* may be the result of an alternate regulatory mechanism that does not operate in homologous cells of *Ciona.* Thus, while the spatial pattern of ddN specification is highly conserved, likely constrained by the invariance of the MG and its developmental lineages, there have been changes to the temporal dynamics of this process. Although the developmental timing of the ddN is identical between *Molgula* and *Ciona* in terms of number of mitotic generations, *Molgula* development is accelerated on an absolute timescale (e.g., hours post-fertilization). This accelerated developmental rate on an absolute timescale may have required very different regulatory strategies simply to maintain the same output (i.e., specification of the A12.239 pair of cells as the ddNs). Although this hypothesis remains untested, it will be interesting to investigate in the future whether the root of the many *cis*/*trans* incompatibilities observed between these geometrically identical embryos lies mostly in their different absolute developmental rates. This would be an extension of the models for how DSD arises, posited by True and Haag [18] and later expanded upon [55–57]. Stabilizing selection would have maintained the same exact MG neuron arrangement, while compensatory changes would be needed to account for the different absolute developmental rates. Unfortunately, the extreme divergence of tunicate noncoding sequences (Additional file 1: Fig. S2) means phylogenetic footprinting is often uninformative when searching for functional transcription factor binding sites in orthologous *cis*-regulatory elements. This makes comparative *cis*-regulatory functional studies very difficult to pursue in distantly related tunicates, such as *Ciona* and *Molgula.*

The remarkable conservation of embryonic cell lineages and geometries between distantly related tunicates has been recently proposed to also underlie the extreme divergence of tunicate genomes [19]. Because gene expression and cell fates are induced by very precise and invariant cell–cell contact events in tunicate embryos, the gene regulatory networks involved in their regulation have been allowed to drift, freed from the constraints imposed by the mechanisms required by larger, more complex and variable embryos. In other words, precision and robustness of gene expression in tunicates arise from the precision and robustness of the embryo itself and not its gene networks. In larger animals (e.g., vertebrates), this precision and robustness can only come from more precise and robust gene regulatory networks instead. Additionally, while the invariant geometry of the tunicate embryo may have served to release the genome from these constraints, it may have imposed a different set of evolutionary constraints. Because of their invariance, tunicate embryos are unable to compensate for errors in cell fate specification and likely experienced strong selective pressure to maintain precise embryo geometries even as some evolved to develop more rapidly on an absolute timescale, like *M. occidentalis*. It is perhaps due to this evolutionary ratchet that *Oikopleura dioica,* the tunicate with the fastest rate of development and most geometrically constrained embryo, also has the most fragmented, highly derived genome as well [58–63]. Future comparative studies among the tunicates promise to shed further light on the fascinating interplay between embryonic form and genetic architecture in evolution.

## Additional file

**Additional file 1.** Supplemental sequences, information, and figures.
