# Supplementary material for: Developmental system drift in motor ganglion patterning between distantly related tunicates
Source: EvoDevo. 2018 Jul 23;9:18. doi: 10.1186/s13227-018-0107-0 (PMC6057086; doi:10.1186/s13227-018-0107-0)
Supplement: Supplementary file 1 — Additional file 1. Supplemental sequences, information, and figures. [file 13227_2018_107_MOESM1_ESM.docx]

**1. Table of *Molgula occidentalis* mRNA *in situ* hybridization probes used in this study**

| Gene | Other name(s) | Cloning method | Vector |
| --- | --- | --- | --- |
| Celf3/4/5 | Etr-1 | RT-PCR | pCRII |
| Dmbx |  | 3’RACE | pCRII |
| Ebf * | COE | RT-PCR | pCiprobe |
| Fgf8/17/18 |  | RT-PCR | pCRII |
| Islet |  | genomic DNA PCR | pCRII |
| Lhx3/4.a * | Lhx3 | 5’RACE | pCRII |
| Mnx | HB9 | 5’RACE | pCRII |
| Neurogenin |  | 3’RACE | pCRII |
| Nk6 | Nkx6 | RT-PCR | pCRII |
| Onecut | HNF6 | RT-PCR | pCRII |
| Pax3/7 |  | RT-PCR | pCiProbe |
| Slc18a3 | VAChT | RT-PCR | pCRII |
| Vsx | Chox10 | RT-PCR | pCRII |

* Probes previously used/published in reference [17]

**2. Predicted probe sequences (SENSE strand, not all verified by sequencing)**

>Celf3/4/5 (Etr-1)

TTGCAGCATTACATGGAAGTCAAACTATGCCTGTAAGTTTTAACTTCTTTATTCAATTTTCTCATTTAAATAAGCGTAAATATAATAATACAGTTAGTTAAATATAGAAAAAAAATGATTAATTCTTGCCAAACACTTTTTTGCTGGAAAAAATCCACATATAAATTTTCCTTAGAGTGTGGCAATCTACACACAAAGGAATCATTTACAATCGTAAATGATGACATAAGAAAAGAAAAGTTATATTCTAAATTATTGTTCTAATAGTCCAATTTTATAATTATGTAAGCAGGAGTAACATGGTAATATTGTAGTGCATTGTGAAGCAAAATTTATGTTATTTTAAATTATACAGTGTACTTGCCTTTATATTTAAATACAATGAAAATTGTCAATACTGCTAATAGTTCTTTGTGTGTTTGAATTTTGAAAAGTGCTTTGAGAATAGCCATAGGTGTAATAAAGTTTGTCAGTTGCCATATGTAATTTTGACCATTAATTATTAAAGACATTTTTTAATTATTTATATCGCTATTAAGTAGATGGAATACTATGATAAAGTTAGTCAACTTAATAATTTAAATTTATTTGCCATAAGGGAGCCTCTTCAAGTTTGGTAGTAAAACTGGCTGACACAGACAAAGAGCGAGCAGTTCGAAAAATGCAGCAGATGGCTAACAACTATGGTATAGTTAGTCCAGTGGCTCTACAACTGGGAACTTACCCAACACATTCTATTGTGGCTGGACCAAGCATGGTACCTTCGGCAGGTTGGTCACCAGTAGCAACAGCATTATCATCAGGGCAATTTGGTCACGTTGCAGCAACAGGCTTAGGGCAAAGTTCGATTGTTCAGTCAAATGGTCCTAGTACAACTCCAGGTATACCAAGTACTCCACAAAGTCCAGTTGCTTCTATCACAGCATTAAATTTGGTTCCACCAACTGTAGTTTCGCAATCTAATGGAATTTCAACTGGAGTATCGCCACAAGAAATTTACTCACTTCCTACATATCCAGGTAGCTCTCAAACTCCACCGGCTGTAGATATGCTCCAACATCCTGTGTACGCACAACATCCACCATACACTGTTGTATATGTTCCATCACAATCATATGGCGGAGGTCAAATTTCAACACCAGGTTTAACCCCAACTGCCACGCCCTTGGGGCCACCACTTACTGCACCACAAGCTGCTGCTATGCTGAGCACAAGTCCAACTGCTCCACAAAAAGAAGGTCCAGAAGGATGCAATTTATTTATTTATCATCTACCACAAGAATTTACTGATGCAGATTTAGCAAATGTTTTCCAACCATTTGGATCCGTAATTTCAGCAAAAGTGTTTATTGATCGCGCTACAAATCAAAGCAAATGTTTTGGTTTTGTTAGTTATGATAACCCATTAAGCGCGCAGACAGCGATTCAAACTATGAACGGTTTTCAAATTGGAATGAAACGCCTCAAAGTTCAGCTGAAACGTCCAAA

>Dmbx

GGAGTCGAACTGCGTTCACCTCCGACCAACTGAAAGCGTTAGAGAGCACGTTTGAAGACACTCAATACCCTGACGTCATCACCAGAGAGAGGCTGGCCATGTTTACAAATCTACCAGAGGCCAGAGTACAAGTGTGGTTTAAAAACAGAAGAGCAAAGTACCGAAAGCAACAAAAACTCTCGAACGGAAAGAGCAAAAATCCGAGCAAAGTGTCGAACAATGAAAACCTCTCTGAAGATGCCGAGTCCTGTAGCAAGACATGCTCTCTTGCTCAAAAAGATCCAGTACATACGGAAGATGATTTTGATAAATCGGATAAAGATGTTGAACGTGTGCAACCAAAAAGTGAAGATGAAACTAAACGTGAAACGCAGGAAATGAAAAATGATTCCTACGTTCCGAAAATACCTGAGCATAAATTTTCGTTCCCGACTACCATGCGCCACCACCTGACTACATACCCAATGCTCCCCTGGTTGTCGTACGCCCACATGATGGCGGCATCCGGTTTGTTACCGGTTCACAACGGCATGGAAAACTTCGGTCCGTTTCCACCAATCCCGTTCCCGCCAACCAATCCAGAGTCGCTCGTCAAAGCTAAAGATGAAGTTCAATAATATTACTTTTCATTTTTTGTATTTAACTTTTTTATTTTATGTCACTGTATAACAATAAAATTCAATCGTTGTTTTTTAACTG

>Ebf (COE)

GCCGGTTTGCATAGAGCTCATTTTGAAAAGCAGCCGCCAAGCAATCTTCGCAAAAGCAACTTCTTTCATTTTGTTTTGGCTTTGTACGATAGGCAAGGTCAACCCGTGGAAGTCGAAAGAACAGCTTTTGTCGATTTCGTTGAAGGGGAACGAGAAGTTGTTACAACTAGTGGCGAGAAAACAAACAATGGAATTCACTACCGTTTGCAGTTACTCTACCACAATGGAATGCGAACGGAACAAGATTTATTCGTTCGGCTTATTGATTCGGTCACAAAACAAGCAATTTCCTACGAAGGACAAGACAAAAATCCTGAAATGAGGAGAGTATTGCTAACGCATGAAATAATGTGCAGCCGGTGTTGTGACAAGAAAAGTTGTGGAAATAGAAATGAAACACCTTCCGACCCTGTTGTGATCGACAGATATTTCCTGAAATTTTTTTTAAAGTGCAATCAAAATTGTTTAAAGAACGCTGGAAATCCCAGAGACATGCGACGGTTTCAGGTTGTTATCTCAACCACAGTTCACGTGGATGGACACGTTCTTGCTGTGTCCGACAACATGTTCGTCCACAACAACTCTAAACATGGGAGAAGAGCGAGAAGGATGGAACCTTCTGACGCAACACCCGTTATTAAAGCATTAAGCCCAAGTGAAGGTTGGACTACAGGCGGCGCAACTGTTATTATAGTGGGCGACAACTTTTTTGATGGATTGCAAGTTGTCTTTGGTTCTATGATAGTTTGGAGCGAGCTGGTGACACAACATGCAATAAGAGTGCAGACACCGCCAAGACATGTGCCTGGTGTAGTAGAAGTAACATTGTCGTATAAGAACAAACAATTTTGCAAAGGTTCTCCAGGAAGATTTGTGTACACAGCCCTGAATGAACCTACTATTGATTACGGGTTTCAGAGACTGTTAAAAGCTATTCCAAGACATCCCGGCGACCCTGAACGTTTACCAAAGGAAATCGTACTAAAGCGAGCGGCTGATGTTATGGAAGCTGTGATGACAAGGTCTTATAACCAAGTCCCGGCGCCTCCTCCAGCTCCTATCCACAACGCATTTAACGGTTCGTCACCATCCATGATGACAGGAAATGTTAATGGTTACAACCACCACATGCCGTCTGCACAATATGGATTAGCAACACCTGATCGACTGGATTCCGCCAACGGTAGCGACTCCGGTAAAATATTTTTAGGAAACAAATTGGGCGGTAAAAATCTCAATTTATATATTTTAAGTATACTTTTATTTTCCAACTAAATCGAAGTATAGTTATAAAATTGTAGCTATTTATCTTTTTTTTATTATCTAGGTTATTCAAGAGGGAGTGCTTCTCCAAAAACTGGTTATTCGCCACAAGGAACACCCCATAGTACTGCCAATGTTGGATTATCTACTGTTGGGGGTAATGTCCCGCCTTATGGCAATGCCATGAATGGGTATTCATGCAATCCCACTTTCACCAATATGACAAATTCAACATCCAACATGTTTACTGGTGGTGGATTGTTTCCAACTTCTCCAAACGCATGTATGAATGCATTGCCAACATGCGGAAGCACACCAGGTATATTTTCATTTTCTCCAGCAAACATGATATCAGCTGTAAAGCAAAAAAGTGCTTTTGCGCCGGTAGTTCGTACTCACAACTCGCCGTCTCCAA

>Fgf8/17/18

TACAGGTAACAGCTGCAGACAGTGTCGATGACTTGTTTCGCAGTCACGTGATCGAAGAGACAAACCGAGGTTCCGATGAGTTGAGCTCACGAAAGACGAGAAATATTCGATTATATTCGAAAGTAACAAGCAAACACGTGCAAATATTAGGAAAAAAAGTCACCGCTTTTGGAGATGATAAAGATCCTTATTCGAAACTTTTGGTGATATCTGATTCACTTGGTGGGCGCGTGAGGATTCGCGGGGAAAAATCAAAATACTATCTTTGTATGACACCGAATGGTCGCGTGGTTGGGAGAAAACGATTTCTCGGCAACCAGTGCACATTTGTTGAGGGTCTGACAGTGAAATATTACAATATATTTTCAAATGCCGCATATCCGAACTGGTATCTGTCGTTCAATCGTCGGGGTCGTCCAAAGAAGGGACCAAAAACAGGTCGAGACGATACAGGAAG

>Islet

GGTGCGCCAATCCAAGATCAATATATACTAAGAGTAGCGCCAAATTTGGAGTGGCACGCCGGATGCCTCAAATGCGCCGATTGTGGCCAAATATTAGACGAAACTAGAACTTGTTTCGTAAGAGATGGTCGGACTTACTGCAAGCATGATTACATAAGGTTGTTTGGGACAAAATGTAACAAATGCGGTTTGGGATTCAGTACTGATGACTTTGTAATGAGGGCGCGAAACAAAATTTACCACATAGACTGTTTCAGATGCGTCGCCTGCAGTCGACAGTTGATTCCAGGAGATGAGTTTGCACTCCGGGAAGAAGAGTTATTCTGCAAGGCTGATCACGATGTTGTAGAAAGGGGGGATGTTATGGGAGTAATGCCAAATGGCGAGATGCCCATGCCAGGTATGTCGTCGCCAATCAGTTTGTCGAACGGAGGGGTTATGAGCCCGGGGGGAATGAGTGGGATGGGAAATTCAGTAATGGGGGGAGGTTTATGTCATAACAATGGCAACACAAGCGGGACATCAAGTGGTAGTTCCCGAAGGTCACACAAAGATCCAAAAACGACGCGAATTCGAACAGTTTTGAACGAAAAACAACTGCATACGTTACGAACATGCTACGCTGCAAATCCGCGACCGGACGCGCTAATGAAAGAACAACTGGTCGAAATGACGAATTTATCGCCGAGGGTCATCCGCGTTTGGTTCCAAAATAAACGCTGCAAG

>Lhx3/4.a

ATTTGGTTGTATTAAGAAATATTAAAACTATTTGTTTGAACTTTGTAAAGTTTGTTTAATCGATATCGAAAACAATGGAGACAGTTTCTTCGAAATGCCTCGGCGAAAACTTTTACACTCGATATGATGATACAAACAAAATGGCGGACAGTGAAATAGACATATCTCGCGAAAATTCTAATCACATGTTTAATGACGTCACAAGTGCGATGTTTGCAGAGGAATGTGACGACACTGAGGTTTATGAAGATGACATCTTGTTTGATAATGATGAAGTCATTAAAACAAACTTATCGTCGTTTGTTGGAAATCACAAGAAAGATTATTTTTCAACTTCCACGCCGCAAAGAGGTACAAAAACAAAACAATCTTCCGGGTTAACAAGCGGGTTACCAGCAAAAATGAATCCTTCGTTAGATAAAAATGTGAACAACAAAAATGTTTTGTACTCCCTTCTAGAAAATGGAAAAAAAATTTCAAATAACTTTTCAAAGTGCACTGGTTGTGGACACCACATATTAGACAGGTTCATACTGAAGGTACAGGATAAACCGTGGCACGCAAAGTGTTTAAAGTGCCACGAGTGTTTGTGCCAGTTATCGGAGAAGTGCTACTCCCGAGATAACTATGTTTTTTGCAAAGAAGACTTTTTTAAAAGATTTGGTACGAAATGTGCTGCGTGTGGACAGGGCGTTCCACCTACGGAAGTTGTGCGAAGAGCTCAAGAAAACGTTTATCATCTCGACTGTTTTTGTTGTTTTATTTGCAACGAGAAAATGGACACGGGAGATCATTTCTATTTAATCGAAGACGGAAGACTTGTTTGTAAAAACGATTACGAGCAAGTCAAAGCGAGAGATATTGATTTTGAAAGTGGATCCAAACGACCAAGGACAACAATATCAGCCAAACAGTTGGAAACTTTGAAGTCAGCCTATAACCAAAGTTCGAAACCGGCGCGACATGTGCGCGAACAACTGAGCGTGGATACAGGGCTTGATATGAGGGTGGTCCAGGTTTGGTTTCAAAATCGACGAGCAAAAGAAA

>Mnx

ACATGGTTGTATTAAGACGTATATTGTTTAAATTAAACTTAATAAAAAGTATATATATTATGATAAGTGCCAGAAATCATTTGCCAGACGCTGTAACTGTACGAAATATGAGCGCAATCATGGCGGCAAATTTATCCAACAGAAACGAACGCAGTTCTTACGAAAGTCCTGTTTACGATTCCAGCGAAGACGATCCGATTCACCCGGAATCCCCGGGGTATCAAAAACGATCAAGTTCGTCATCTTCTTTGGCGTCCTTATCACCATTGTCTTCCTCGCCACAGCACTTTCGTAACGAGAATGTAAGATCGTCATTGGAAGAAGAGGCTGTAAATCTTTCCTCAAAGTGTTCTAAATCCACAGATTGGGATTCAAAAAAATGCAATGGACTGAAAAAATGTTTAAAAAGTTCCAAATTTGGAATTGATGCAATCCTAGCGTCTCCTTCTTCATCCCAAGCAAGTAAAAACTCTTCAGATCAAAACGGTTTAAAAATTAAACGATCTGCAAGCAACGAGGGTTGGAATTACTTTTATCCGCGACAAAATACAACCTCAGAATCTCGTAAAATACAAAATGGCGATAGCGATTCCATTAAAAGATTATGTCCTTCCACCGATAAAGACACTTCTTTAATCGACGTAGTCAATGCTAGCCCAGTAAGTTCAAAGTCCTATGAAAACAGTGACCACCACGTATCACCTACATTGTATTCTGGAATTCCCAAACCCCATATTGATGCTTTTCAAACCTTTTTCCACCCAAAAAGAAGATCATTCGAACATGACGGAATCGAATCAACAAATAAATCTGAAGATGCTAGATTAGAGACTGCTATTCCACCAATGGGACTGGGTCAGCAGTATCATGGAATGCATATTAACCAATATGCAATTGCAGCTGCAATCGCTGCAGCCGGGTTTGAAAGAAATCAACTGAGTTCTTATGCAGTTCCGACTAATATTCAACAAAATACAGATGAAAAAACTTTTCGTAACGAAAGCAATGATAAAGATTACACAATGGACCCATCCCAACATTATCCAAGTTTACCCCAAAACCCAGCGTTGTACTACCCCGGTAGTATATCCCCGATGTTACCAGGGCACCCATCGTTTTTTGGAAAATACCAATTTCCCCAACCGGCATGTACAGGAATGCCTGCACCAATGCAGCACGTGGGAATGAGCAATCCAACAGCGCAACAACAAGTAGTCGCTATGGAAATGTTAAGAAGTGGTCGCATGTTTCGGGACATCACAGAATTTGCAGGTCATGTTCATCCGGGTCTCTTAAGTCGTTCCAGA

>Neurogenin

AGCTCCGAAGAGGAAAGGCAAACGACCGTGAAAGAAATCGTATGCATGGGCTAAACGATGCTCTCGAAAATTTAAGGAGAGTATTGCCAACTTATCCTGATGAAACTAAACTTACAAAAATTGAAACTTTGCGATTCGCTTACAATTATATCTGGTGTCTCAGCGAATTAGTCAAGTCCGGCGATTCTGGAACAGATAAATCACAAAATGGTGCACCAAATCCTTTTGAAATAAACGATGACCAATATATTGACAACACACAAAGCGAAATAAATCAAATGACACCAACTGCCGGTCCACAGCACATGTACCAGGCTCAGTACCAAGGTCAGCCACCCATGCACCACTCCCAATATTATGATGCGTACAACCCAGAACAAATAGCATCTCCAAATTCATTTGGAAACCAAGCCAGTCAAATAAATGGTGGGATCGATTGCTTCGATGAATTTGAGGATATCCATCAGCCATTTATTACACAGCAATATAACCAAATTCCACGACAAGAAGATTTGAAATGCAATAATTATCAGGATGTTAACTACGCATATTCACCAGCAAACAGTAACAACAACAACAACAATTCTGTTGTACCTGTGCATACAACATCAATTGGTTACAGGCGAAATGCGAAAGAAATTACACTGAATGGATTAAGTCAGATACAACACCCGTCTCCACTAACCCCTCCCATGTCGGCTTCCTCTCCCCATAACCAACAAAGCCCGTTTACGTTACTTCCACAAGAGCAACAACACTTTACATTTGTACCACCTCCAACTCCAAGCGACACCGGTAGTCTTTCTAGTGGTTCTCCGTTTAGTAGCCCCCAAAAGCTGTTGGCAATCACCGACGGAAGGTGTCAGCGACCGACCGAGATGACATATGCCACCAACTCTCAAGTGCCAGCCACTGTAAACCAGCATAGTTATACTCAAAGTACTGGAAATTATTTTTTACTTCATTAATATTTGCGNGACATTTTGGTGCGTGTTAAACATTCACCATTTTGTTCATTAGAAATATTTAAATTATTTCTTCCATATTATTATTATTGTTCTATAAGTTGTCTTTTCGTACTGTCTTGTTATTATAATTATTACTATTATTATTATTATTTAATCCTTTATTATCCTCTGTTGGATATAAATGATTGGTAACAGACTAGAATGTTAAATTTAATCGATACTGAAAGAGCAAAGTTTGCTTCGCATAATTAGTTCTTCAATACCATTTTTTACATAAATAATGTGTTCGTTTGTCAGTTTGTGATTTATACTTTACTGCCAACTTTTTTGATCTTCTTCATATACTGCCATTGTAAACCCTCTGCCTTTTGACACCGCTTCTTTTGAACTGTTTTGTTAGTAATTTTATTATGCAAATAAAAATCCCAAAAC

>Nk6

GGGACTCCATTTGGAATAAACGACATTTTAAATCGACCCAGCACTTCAAGCGACGTTTCGTCAAATAGTTCCAGCCCGCCCAGCGCGAATAGTTCTAATACCCCCGTTGGATATTTCCCTGGACCCCACGTGCCTGTGACGTCACCGGGGGCAGCAATAGCAGCCGCAGCAGCCATGTACTTAGGAGGAGGGTCAATGGGAGCAACATCTGCCGTCCCCGGGACCATGCTGAGTCAAAGTCACATGGGGCGGTTTTCAAAACCCTTGGCTGAACTTCCCGGTCGCTCGCCCATCTACTGGCCAGGAGTATTGCAAAAAGATTGGCAGGAAAAGTTCTCTTGTCAAGCGCACCCAGGTTTGATTGTGGATAAATTTGGAAAGAAAAAACACACGCGCCCAACTTTTTCCGGCCAACAAATTTTTGCACTTGAAAAAACTTTTGAACAGACGAAATATCTGGCAGGTCCTGAAAGAGCGAGATTGGCGTATTCGTTAGGGATGACGGAATCA

>Onecut

AAGCTATGCAACATTGACACCGTTACAACCACTTGCTTCTTTAACAACAGCAAGTGATAAATATATACCGGTAAGCACCAGTCCTAATTTCTCGTTAGTAAATCCATCTGATGTCTCAGGCTTGGGTGACATTAATGGAAATTATCAAAAAATGACTGGTATGGGCCAAAGTCTTCCACCATTATCAAACAGTATGTTAATCGGTGGTTTGTCAACCGGAACGGACAGTATTCATCCTCCATCAACTACTTTAACTTCTAATGTTCAACCCGAATCCACAATAAGCACTGGTGGAATGCATCTACCGCAATATGCTCGATCGCCTAATAATTTTACAGGAACTAATTCGTACGATTCCCGTTTATTCGACGCTGCTTCTGATACGTTCACCAACCCCCAGTTATCCGCCACCCCAATGTTTTCAAGACGAAGCTCCGGGTTCACAACTCCCCATTCTATCAACGCATCATGTTCTCCCATCAACTCGCGTGTTAACAGAGTGCCCAGGGGATCACCAGTCAATCTAAATAGCGCGAATCAACGTCAACAAAATAACGAGGAAGTTAACACTAAAGAAGTAGCCGCGAAAATAACACAAGAATTGAAACGATACAGTATTCCTCAAGCTATTTTTGCCCAACGCGTTTTATGTCGAAGCCAAGGTACCTTGTCGGACTTATTG

>Pax3/7

GGGCGAGTAAATCAGCTTGGGGGAGTGTTTATAAACGGCCGACCTTTGCCGAATCATATTCGCCACAAAATCGTAGAAATGGCGGCGCATGGTGTACGACCTTGCGTTATCAGTCGTCAACTTCGCGTCTCTCATGGATGTGTCAGTAAAATCCTTTGCAGATACCAAGAAACTGGTTCGATAAAACCGGGTGCAATTGGAGGAAGCAAACCCAAACCCAACAATAGTGATATTGACAAAAAGATCGAGGAATATAAAAAAGAAAATCCTTCGATGTTCAGTTGGGAAATCAGAGACAAACTCATAAAAGAAAATATTTGCGAAAGAAGTTCTGCACCAACTGTAAGTGCTATCAGCAGAATTCTGCGCGCAAAAGGATGCGATATTTCCAACGAATCAGAAAACCATTCCAACTCAGACGGAGGTGATGCTGAAGATAGATCAGTGGACGGATGTCAGGACTCAGACTGCGAATCAGAACCAGATCTTCCTTTAAAGAGGAAGCAAAGACGAAGCAGGACTACTTTTTCCGCTGATCAACTTGAAGAACTGGAAAGATGCTTTGAAAGAACACATTATCCTGATATTTACACAAGAGAAGAACTAGCACAAAGAACAAGATTAACGGAGGCTAGAGTACAGGTATGGTTTAGCAACAGACGAGCAAGGTGGCGTAAACAAATGGCTGCACAGCAACTACCAGGATTACATCACCATCCTCATCTTCAACACCATATTGGNTACCATTCCATAGCAGCTGCAGGAATGTCGGCTCACAATTACATGCTT

>Slc18a3 (Vacht)

ACATGGTGATAGTTCCAATTGTGAATGATTATTTTAAAACTGAGAATGAAACAAAGATTTTAACAACTATATCACCGTCTCCATCTACATTAATTAATTCATCCCAGCTACCTCAAGGCCTTATGAGGCATCTTCAGCAAGAAGCACATTCTCGTGGCCCCTATGATAGATACGGACCCGAAGCCCATCGAAGGCATCAAAATAAAATTTTAAAGTTAGCTCCTCCACATGAGGAAAGCACTAAAAATGGAATGATGGGTGTGTTATTCGCATCCAAAGCAATTGTCCAGCTTTTAGCTAACCCATTTACTGGTGCTTTTATCGATAGAGTTGGATATGTAATGCCTCTAACCATGGGGTTAATGGTAATGTTTGTTTCAACCACTGTATTTGCGGCTGCTTCGTCTTATACTTGGCTATTTGTCGCGAGAAGTCTCCAAGGTTTAGGATCTGCTCTTGCTGATACAGCGTCATTTGGCTTTATTGCTGATCGATTCACGGACGAAAAAGAACGGTCAACAGCATTGGGGATAGCATTGGCTTTTATTTCCTTTGGAAGTTTAGTAGCACCTCCATTTGGAGGGGTTTTGTATGAGTTTGCGGGCAAAATGTGGCCATTTCTAATTTTGGCGTTTGTATGTTTCTTGGATGCAATGTTGCTCCTTTTAGTTGAAATTCCAACGAAAAAAGAAGATGGAATGGAAGCCGCTAAAAACCAGCCAGGAACGCCGATCTACAAGTTATTTGTCGATCCGTATATAGCTGTAATTGCAGGAAGTTTGATGATCGCAAATTTTCCGCTTGCGTTTTTGGAACCGACCATAGCTGAGTGGATGGAAATGAGAATGCATGCCACTAAATGGCAAATTGGACTAGTTTGGCTCCCTGCTTTTATACCGCACGTCATTGGGGTGTATGTAACCGTAAAAATAGCGCAAAAGTACCCACAATATCAATGGCTAGTTGGAGGATTGGGTCTCGTAATTATCGGAGTTTCAACCTCAGTAGTGTCGGTTTGCACAACATATGAAGTTCTATTCTTACCGCTTGCTACCTTGTGCTTCGGAATCGCTCTTATCGACACGGCACTTTTGCCAACTTTGGCGTTTTTGGTGGACGTCAGACACACAAGTGTATATGGAAGTGTCTATGCCATAGCCGATATTTCGTACTCGGTGGCTTATGCCTTAGGGCCTGTACTCGCCGGACAAGCGGTTCAAGTTTTGGGTTATCTGAAGATGAACGTGACGATCGGAATTGCAAATATGATGTTCGCCCCGGCTTTACTATTTTTACGAAACGTTTACGACTGGAACA

>Vsx

ACTTCGCAAAGAAAATCCGTGGTTGTTTTATCAACCTTATCGTCGTTTGAATGAAACTTACGAAAACTTTTTTTACCAACTAAATGCAAGTGTTCAGAAAATTGAAAAACAAGAAAGACCGGAGTTCTTAAAACTAGAAAATTTGAATAAAAGTGAGAATCAAACTCAACAAGAAGTCGTTGATTCCGTAAAATTGACCCCAAAATCAAAAACAGATTGCACAAAAGCGAACAATCTAAAAACACTAAAATTGGAAATTTCAAATCGCCTGAAAAAACGAAGGAACAGAGCGGCATTTAATGAAGAACAAATAAAGGCATTGGAAGACGCTTTTGAAGCAGCTCACTATCCGGACATTGTCGCCAGAGAGAAACTTTCATTATCAACCGGCATTGAAGAAAGCCGCATACAAGTGTGGTTTCAAAACAGACGTGCAAAATGGAGAAAAACAACTAATGATTGGGGTCACAGTAGTGTTATGGCTGAATATGGTTTATATGGAGCCATGGTGCGGCACTCCATTCCCTTGCCAAATAG

**3. pCiProbe *in vitro* transcription vector**

M13R primer sequence

T3 promoter sequence (not used)

T7 promoter sequence (used for *in vitro* transcription antisense probe)

M13F primer sequence

AmpicilinR

NotI restriction enzyme site

EcoRI restriction enzyme site

CAGGAAACAGCTATGACcatgattacgccaagcgcgcAATTAACCCTCACTAAAgggaacaaaagctggagctccaccgcggtggcggccactagtataGCGGCCGCataGAATTCatagctgagcataggtacccaattcgCCCTATAGTGAGTCGTATTAcgcgcgctcACTGGCCGTCGTTTTACcaacgtcgtgactgggaaaaccctggcgttacccaacttaatcgccttgcagcacatccccctttcgccagctggcgtaatagcgaagaggcccgcaccgatcgcccttcccaacagttgcgcagcctgaatggcgaatggaaattgtaagcgttaatattttgttaaaattcgcgttaaatttttgttaaatcagctcattttttaaccaataggccgaaatcggcaaaatcccttataaatcaaaagaatagaccgagatagggttgagtgttgttccagtttggaacaagagtccactattaaagaacgtggactccaacgtcaaagggcgaaaaaccgtctatcagggcgatggcccactacgtgaaccatcaccctaatcaagttttttggggtcgaggtgccgtaaagcactaaatcggaaccctaaagggagcccccgatttagagcttgacggggaaagccggcgaacgtggcgagaaaggaagggaagaaagcgaaaggagcgggcgctagggcgctggcaagtgtagcggtcacgctgcgcgtaaccaccacacccgccgcgcttaatgcgccgctacagggcgcgtcaggtggcacttttcggggaaatgtgcgcggaacccctatttgtttatttttctaaatacattcaaatatgtatccgctcatgagacaataaccctgataaatgcttcaataatattgaaaaaggaagagtATGAGTATTCAACATTTCCGTGTCGCCCTTATTCCCTTTTTTGCGGCATTTTGCCTTCCTGTTTTTGCTCACCCAGAAACGCTGGTGAAAGTAAAAGATGCTGAAGATCAGTTGGGTGCACGAGTGGGTTACATCGAACTGGATCTCAACAGCGGTAAGATCCTTGAGAGTTTTCGCCCCGAAGAACGTTTTCCAATGATGAGCACTTTTAAAGTTCTGCTATGTGGCGCGGTATTATCCCGTATTGACGCCGGGCAAGAGCAACTCGGTCGCCGCATACACTATTCTCAGAATGACTTGGTTGAGTACTCACCAGTCACAGAAAAGCATCTTACGGATGGCATGACAGTAAGAGAATTATGCAGTGCTGCCATAACCATGAGTGATAACACTGCGGCCAACTTACTTCTGACAACGATCGGAGGACCGAAGGAGCTAACCGCTTTTTTGCACAACATGGGGGATCATGTAACTCGCCTTGATCGTTGGGAACCGGAGCTGAATGAAGCCATACCAAACGACGAGCGTGACACCACGATGCCTGTAGCAATGGCAACAACGTTGCGCAAACTATTAACTGGCGAACTACTTACTCTAGCTTCCCGGCAACAATTAATAGACTGGATGGAGGCGGATAAAGTTGCAGGACCACTTCTGCGCTCGGCCCTTCCGGCTGGCTGGTTTATTGCTGATAAATCTGGAGCCGGTGAGCGTGGGTCTCGCGGTATCATTGCAGCACTGGGGCCAGATGGTAAGCCCTCCCGTATCGTAGTTATCTACACGACGGGGAGTCAGGCAACTATGGATGAACGAAATAGACAGATCGCTGAGATAGGTGCCTCACTGATTAAGCATTGGtaactgtcagaccaagtttactcatatatactttagattgatttaaaacttcatttttaatttaaaaggatctaggtgaagatcctttttgataatctcatgaccaaaatcccttaacgtgagttttcgttccactgagcgtcagaccccgtagaaaagatcaaaggatcttcttgagatcctttttttctgcgcgtaatctgctgcttgcaaacaaaaaaaccaccgctaccagcggtggtttgtttgccggatcaagagctaccaactctttttccgaaggtaactggcttcagcagagcgcagataccaaatactgtccttctagtgtagccgtagttaggccaccacttcaagaactctgtagcaccgcctacatacctcgctctgctaatcctgttaccagtggctgctgccagtggcgataagtcgtgtcttaccgggttggactcaagacgatagttaccggataaggcgcagcggtcgggctgaacggggggttcgtgcacacagcccagcttggagcgaacgacctacaccgaactgagatacctacagcgtgagctatgagaaagcgccacgcttcccgaagggagaaaggcggacaggtatccggtaagcggcagggtcggaacaggagagcgcacgagggagcttccagggggaaacgcctggtatctttatagtcctgtcgggtttcgccacctctgacttgagcgtcgatttttgtgatgctcgtcaggggggcggagcctatggaaaaacgccagcaacgcggcctttttacggttcctggccttttgctggccttttgctcacatgttctttcctgcgttatcccctgattctgtggataaccgtattaccgcctttgagtgagctgataccgctcgccgcagccgaacgaccgagcgcagcgagtcagtgagcgaggaagcggaagagcgcccaatacgcaaaccgcctctccccgcgcgttggccgattcattaatgcagctggcacgacaggtttcccgactggaaagcgggcagtgagcgcaacgcaattaatgtgagttagctcactcattaggcaccccaggctttacactttatgcttccggctcgtatgttgtgtggaattgtgagcggataacaatttcaca

**4. *Molgula oculata* *Dmbx* reporter construct sequence**

**(Moocul = *M. oculata)***

*Cis-*regulatory DNA

ATG = predicted endogenous start codon

Unc-76::eGFP

SV-40 polyA signal

AmpicilinR

>Moocul.Dmbx -1390/+24 > Unc-76::eGFP

ggcgcgccTAGGTCAAGACCTAAGACACCAAACTGCTGACCAATCTGTTTTCAAATCGACTTTTTGTTTCGTGAATAAAATACAATTAATTTGTGAAATCCAATGAAAAGTAATCGTTTTGTCGTTCTGTGATTAATTAAATACATAATTAATAATTAATTCCTCTTACTCTTGAAGTAAATCAGAGAAGTGATCTTAAACTTATCCACCGTGACCATTAACTCTGTCTGTTTAAAGATTTTTATGCACACACTTGGCATAAGTTAATTAATTTGTCGTATCTTTTTAATTAGCTATTCAATTGTCGTGATAGATGGTGAATGGAGCTAATAGGAAATGGTATTTCGGAACATATCGGCCACTAAATAACCATGAAATTACTGAATATTCTATAAAATCTTGTGGTATAAAAGCTGAAGGAACACGAAAGTTTAACGACCCGTATTAACAGGCAAAGATTAGTTGGCACCTGTACAGAGTACACGGTGGTGGGTCGGTTATGTAAAAAACCTGTGGTATATCTAANGGATTAACTAAATTTCGTAATATGGATTACGCGAGAACTAAAAGCATTATAACCTATTTTATTTAACTCCTTGAAGCGACTTAGTCCCGTGCGATCTCGGAACCAAATATACATTTTATTTATCTTGTTCAGAAGAGATGTTATTGAAGCGTTTCATAATATCCAGTTAATCGGATTAGGCAGAAGGTCTTACACAAAAGCACCTGCGAAAAAATGTTCGTTGAAAAGGATGAATGCCGAACACCTTCCCNAAGTAAACCGAATATTTAGAAAAACATCACCGGATTAAGTCGCAGTTTGTTTAACTCGTGTTTTGAGCTCGACTGACAATCCGGGAAGGATATCTTAATGCCAAAATTGTGAAATAATGCCTCCAATTATCGCTCAACACAAAATGAGTATTAGGTAATTTTCAGCCGTTATTTTATGTCTTTTCTTTTGGCTTAATGGCAATCGGCTTAGAACTAGAATATTTTAAAAATTAATGTGTATCCGTAGCCCTCAAACATTTTGTTGTCAGAAAGTTTCAAAAATATTTTTACAGGCTTATACTGTGGAGATTAGAGCTGACTAATCCACCCTTAAGCACCGCTGGGATCCACTTTTTTTTATATAACAGAGTATTTATAAAGCCATTTGCGATCCGCAGTTAGACGATGAGAGATGACCTTCTATGAACACAGCACGTTCAAGCTTCAGGCATAAAGATATGTTATTTCTGCATTTAGACGGATTTAAATAGTTGCCAACTTAGAGGAGGCGGAAGAAAACGAAGAGCTAATCCAATTATTTTGTGATTTCAACGTTGTTTTACAGTGAGGTCAAAATAATATATTTTTATTATTATTTCCACGTGCTTGAAATAAGGAAAATGAACTCGGATTTGTTCTACAAGgcggccgcaaccATGGCGGATCTGCGAGTACCGGACATTCCGCTCGCCTCGTGTGATGATGATGATATCGATAGTAATAAGAATTTGAGCAACCATTCATCAGACGAGAAACATCACTGCAACAGCAACAGCGACGAGGAACGTCTTCATGACGAGTTCTCTGGATCCCTTGAGGACCTTGTCGGCAACTTTGACGAAAAAATTGCGGCATGCCTGAAGGACCACGAGGTGACGACAGCGGATATTGCACCTGTGCAGATACGTACTCAAGAGGAAGTTATGAATGAAAGCCAAACATGGTGGACATTAACCGGAAACTTTGGAAACATTCAACCTCTCGACTTTGGAACCTCTTCGATATGTAAAAAGATGGCCGCAGCTCTGGACAGTGATTCATTGAAAGACGACGCATCTACACGCCGAAGTATGACAAATTCCGATGATGAGGATCTTTTACGACAACAAATGGATGTTCATCAAATGATTGGACATCATCATGGATCTACGGATACTGGTGGTGAAACACCTCCACAGACTGCTGATCAAGTTATCGAAGAAATTGATGAAATGTTACAGGTACCGGTCGCCACCATGGTGAGCAAGGGCGAGGAGCTGTTCACCGGGGTGGTGCCCATCCTGGTCGAGCTGGACGGCGACGTAAACGGCCACAAGTTCAGCGTGTCCGGCGAGGGCGAGGGCGATGCCACCTACGGCAAGCTGACCCTGAAGTTCATCTGCACCACCGGCAAGCTGCCCGTGCCCTGGCCCACCCTCGTGACCACCCTGACCTACGGCGTGCAGTGCTTCAGCCGCTACCCCGACCACATGAAGCAGCACGACTTCTTCAAGTCCGCCATGCCCGAAGGCTACGTCCAGGAGCGCACCATCTTCTTCAAGGACGACGGCAACTACAAGACCCGCGCCGAGGTGAAGTTCGAGGGCGACACCCTGGTGAACCGCATCGAGCTGAAGGGCATCGACTTCAAGGAGGACGGCAACATCCTGGGGCACAAGCTGGAGTACAACTACAACAGCCACAACGTCTATATCATGGCCGACAAGCAGAAGAACGGCATCAAGGTGAACTTCAAGATCCGCCACAACATCGAGGACGGCAGCGTGCAGCTCGCCGACCACTACCAGCAGAACACCCCCATCGGCGACGGCCCCGTGCTGCTGCCCGACAACCACTACCTGAGCACCCAGTCCGCCCTGAGCAAAGACCCCAACGAGAAGCGCGATCACATGGTCCTGCTGGAGTTCGTGACCGCCGCCGGGATCACTCTCGGCATGGACGAGCTGTACAAGTAAgaattccagctgagcgccggtcgctaccattaccagttggtctggtgtcaaaaataataataaccgggcaggccatgtctgcccgtatttcgcgtaaggaaatccattatgtactatttaaaaaacacaaacttttggatgttcggtttattctttttcttttacttttttatcatgggagcctacttcccgtttttcccgatttggctacatgacatcaaccatatcagcaaaagtgatacgggtattatttttgccgctatttctctgttctcgctattattccaaccgctgtttggtctgctttctgacaaactcggaacttgtttattgcagcttataatggttacaAATAAAGCAATAGCATCACAAATTTCACAAATAAAgcatttttttcactgcattctagttgtggtttgtccaaactcatcaatgtatcttatcatgtctggatcgacaaagtcaaagcggccatcagatctgccggtctccctatagtgagtcgtattaatttcgataagccaggttaacctgcattaatgaatcggccaacgcgcggggagaggcggtttgcgtattgggcgctcttccgcttcctcgctcactgactcgctgcgctcggtcgttcggctgcggcgagcggtatcagctcactcaaaggcggtaatacggttatccacagaatcaggggataacgcaggaaagaacatgtgagcaaaaggccagcaaaaggccaggaaccgtaaaaaggccgcgttgctggcgtttttccataggctccgcccccctgacgagcatcacaaaaatcgacgctcaagtcagaggtggcgaaacccgacaggactataaagataccaggcgtttccccctggaagctccctcgtgcgctctcctgttccgaccctgccgcttaccggatacctgtccgcctttctcccttcgggaagcgtggcgctttctcaatgctcacgctgtaggtatctcagttcggtgtaggtcgttcgctccaagctgggctgtgtgcacgaaccccccgttcagcccgaccgctgcgccttatccggtaactatcgtcttgagtccaacccggtaagacacgacttatcgccactggcagcagccactggtaacaggattagcagagcgaggtatgtaggcggtgctacagagttcttgaagtggtggcctaactacggctacactagaaggacagtatttggtatctgcgctctgctgaagccagttaccttcggaaaaagagttggtagctcttgatccggcaaacaaaccaccgctggtagcggtggtttttttgtttgcaagcagcagattacgcgcagaaaaaaaggatctcaagaagatcctttgatcttttctacggggtctgacgctcagtggaacgaaaactcacgttaagggattttggtcatgagattatcaaaaaggatcttcacctagatccttttaaattaaaaatgaagttttaaatcaatctaaagtatatatgagtaaacttggtctgacagttaCCAATGCTTAATCAGTGAGGCACCTATCTCAGCGATCTGTCTATTTCGTTCATCCATAGTTGCCTGACTCCCCGTCGTGTAGATAACTACGATACGGGAGGGCTTACCATCTGGCCCCAGTGCTGCAATGATACCGCGAGACCCACGCTCACCGGCTCCAGATTTATCAGCAATAAACCAGCCAGCCGGAAGGGCCGAGCGCAGAAGTGGTCCTGCAACTTTATCCGCCTCCATCCAGTCTATTAATTGTTGCCGGGAAGCTAGAGTAAGTAGTTCGCCAGTTAATAGTTTGCGCAACGTTGTTGCCATTGCTACAGGCATCGTGGTGTCACGCTCGTCGTTTGGTATGGCTTCATTCAGCTCCGGTTCCCAACGATCAAGGCGAGTTACATGATCCCCCATGTTGTGCAAAAAAGCGGTTAGCTCCTTCGGTCCTCCGATCGTTGTCAGAAGTAAGTTGGCCGCAGTGTTATCACTCATGGTTATGGCAGCACTGCATAATTCTCTTACTGTCATGCCATCCGTAAGATGCTTTTCTGTGACTGGTGAGTACTCAACCAAGTCATTCTGAGAATAGTGTATGCGGCGACCGAGTTGCTCTTGCCCGGCGTCAATACGGGATAATACCGCGCCACATAGCAGAACTTTAAAAGTGCTCATCATTGGAAAACGTTCTTCGGGGCGAAAACTCTCAAGGATCTTACCGCTGTTGAGATCCAGTTCGATGTAACCCACTCGTGCACCCAACTGATCTTCAGCATCTTTTACTTTCACCAGCGTTTCTGGGTGAGCAAAAACAGGAAGGCAAAATGCCGCAAAAAAGGGAATAAGGGCGACACGGAAATGTTGAATACTCATactcttcctttttcaatattattgaagcatttatcagggttattgtctcatgagcggatacatatttgaatgtatttagaaaaataaacaaataggggttccgcgcacatttccccgaaaagtgccacctgacgtctaagaaaccattattatcatgacattaacctataaaaataggcgtatcacgaggcccttacgtattaattaa


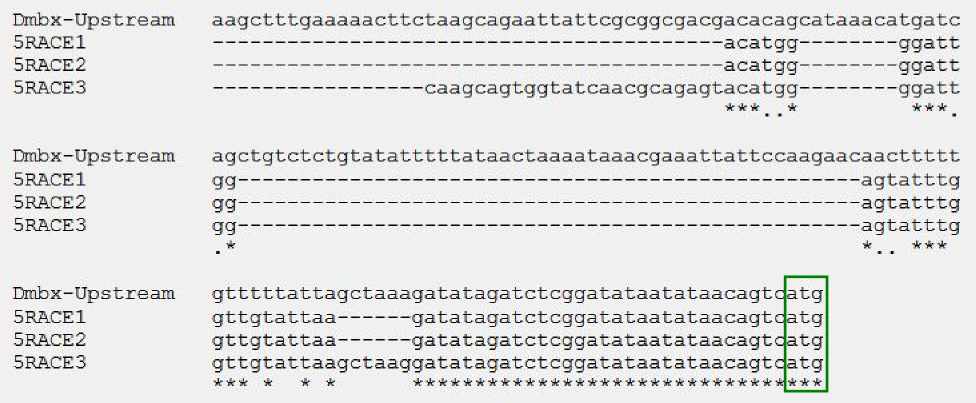


**Supplemental Figure 1. *Molgula occidentalis* *Dmbx* 5’ RACE showing evidence of mRNA trans-splicing**

MAFFT (https://mafft.cbrc.jp/alignment/server/) alignment of three independently cloned *M. occidentalis Dmbx* cDNAs (5RACE1-3, amplified using the Clontech SMARTer 5’ RACE kit) and the immediate upstream sequence from the *Dmbx* locus determined by genome sequencing and verified by cloning (“Dmbx-Upstream”). The cDNA sequences match the genomic sequence up to 30 bp upstream of the start codon of the gene (green box), but have additional 5’ sequences that do not align to the *Dmbx* locus, indicative of *trans*-splicing of a 5’ splice leader, a phenomenon that is widespread in *Ciona robusta.*


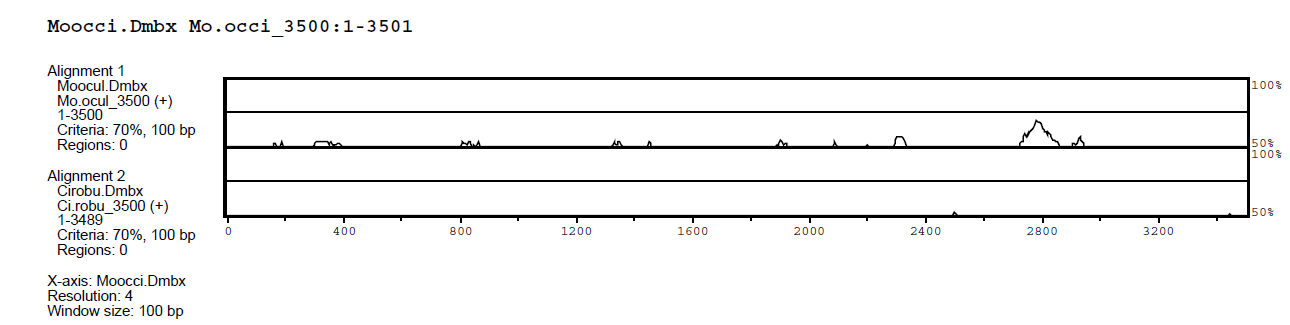


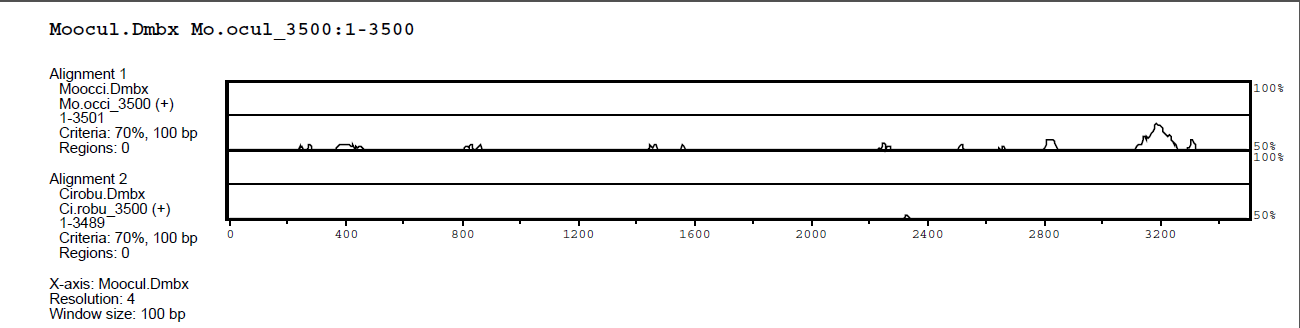


**Supplemental Figure 2. VISTA alignment plot of *Dmbx cis*-regulatory sequences**

TOP: VISTA plots (http://genome.lbl.gov/vista/customAlignment.shtml) of *Molgula occidentalis Dmbx* 5’ upstream region (~3500 bp upstream of the ATG start codon) aligned to the corresponding region from *Molgula oculata Dmbx* (“Alignment 1”) and *Ciona robusta Dmbx* (“Alignment 2”), showing very little non-coding sequence conservation between the two *Molgula* species, and no conservation between *M. occidentalis* and *C. robusta.* The peak of moderate *Molgula-*specific conservation (<70% identity) around position 2800 does not contain the minimal *Dmbx* enhancer (data not shown), and likely represents proximal promoter sequences. BOTTOM: Similar VISTA plots using *M. oculata Dmbx* upstream region as a reference instead, showing similar complete lack of conservation with the corresponding region in *C. robusta* (“Alignment 2”).
